# ROS-responsive liposomes with NIR light-triggered doxorubicin release for combinatorial therapy of breast cancer

**DOI:** 10.1186/s12951-021-00877-6

**Published:** 2021-05-11

**Authors:** Hanxi Yi, Wangxing Lu, Fan Liu, Guoqing Zhang, Feifan Xie, Wenjie Liu, Lei Wang, Wenhu Zhou, Zeneng Cheng

**Affiliations:** 1 Division of Biopharmaceutics and Pharmacokinetics, Xiangya School of Pharmaceutical Sciences, Central South University, Tongzipo road 172, Changsha, 410000 China; 2 Neurology department, The First affiliated Xiangya hospital, Central South University, Changsha, China; 3 Xiangya School of Pharmaceutical Sciences, Central South University, Changsha, China

**Keywords:** Reactive oxygen species response, Prodrug, Synergistic therapy, Photodynamic therapy, Photothermal therapy

## Abstract

**Background:**

Reactive oxygen species (ROS)-responsive drug delivery systems (DDSs) are potential tools to minimize the side effects and substantially enhance the therapeutic efficacy of chemotherapy. However, it is challenging to achieve spatially and temporally controllable and accurate drug release in tumor sites based on ROS-responsive DDSs. To solve this problem, we designed a nanosystem combined photodynamic therapy (PDT) and ROS-responsive chemotherapy.

**Methods:**

Indocyanine green (ICG), an ROS trigger and photosensitizer, and pB-DOX, a ROS-responsive prodrug of doxorubicin (DOX), were coencapsulated in polyethylene glycol modified liposomes (Lipo/pB-DOX/ICG) to construct a combination therapy nanosystem. The safety of nanosystem was assessed on normal HEK-293 cells, and the cellular uptake, intracellular ROS production capacity, target cell toxicity, and combined treatment effect were estimated on human breast cancer cells MDA-MB-231. *In vivo* biodistribution, biosafety assessment, and combination therapy effects were investigated based on MDA-MB-231 subcutaneous tumor model.

**Results:**

Compared with DOX·HCl, Lipo/pB-DOX/ICG showed higher safety on normal cells. The toxicity of target cells of Lipo/pB-DOX/ICG was much higher than that of DOX·HCl, Lipo/pB-DOX, and Lipo/ICG. After endocytosis by MDA-MB-231 cells, Lipo/pB-DOX/ICG produced a large amount of ROS for PDT by laser irradiation, and pB-DOX was converted to DOX by ROS for chemotherapy. The cell inhibition rate of combination therapy reached up to 93.5 %. After the tail vein injection (DOX equivalent of 3.0 mg/kg, ICG of 3.5 mg/kg) in mice bearing MDA-MB-231 tumors, Lipo/pB-DOX/ICG continuously accumulated at the tumor site and reached the peak at 24 h post injection. Under irradiation at this time point, the tumors in Lipo/pB-DOX/ICG group almost disappeared with 94.9 % tumor growth inhibition, while those in the control groups were only partially inhibited. Negligible cardiotoxicity and no treatment-induced side effects were observed.

**Conclusions:**

Lipo/pB-DOX/ICG is a novel tool for on-demand drug release at tumor site and also a promising candidate for controllable and accurate combinatorial tumor therapy.
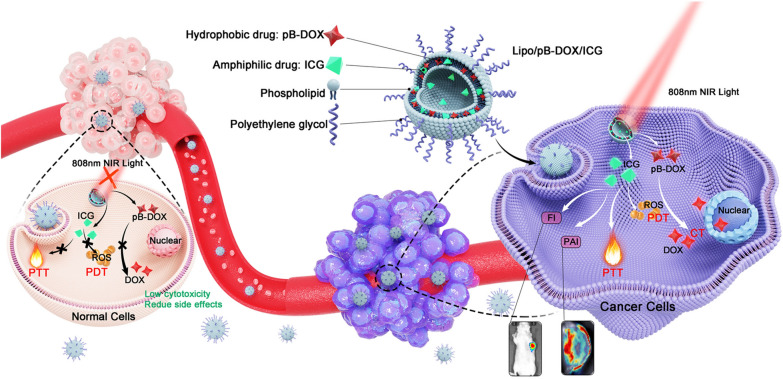

**Supplementary Information:**

The online version contains supplementary material available at 10.1186/s12951-021-00877-6.

## Introduction

Chemotherapy, as a mainstay in clinical cancer treatment, shows severe systemic toxicity resulting from the nonspecific drug distribution in the human body [1, 2]. To this end, extensive studies have been conducted to minimize the side effects of chemodrug by designing various drug delivery systems (DDSs), especially stimuli-responsive DDSs in the past decade [3–5]. Stimuli-responsive DDSs release or activate anticancer drugs selectively in response to an internal stimulus in tumor microenvironments (TME) such as pH [6–8], glutathione (GSH) [9, 10], enzymes [11–13], reactive oxygen species (ROS) [14], and their combinations [15–17]. Because both cancer and normal cells have endosomes/lysosomes with acidic pH [18] and high intracellular concentrations of GSH (2 × 10^− 3^ to 10 × 10^− 3^ M) [19], the widely reported pH- and GSH-based responsive DDSs offer limited selectivity. Instead, hypoxia significantly increases the ROS levels in tumor tissues, making the ROS levels much higher in cancer cells (up to 100 × 10^− 6^ M) than in normal tissues (≈ 20 × 10^− 9^ M) [20]. Currently, a series of ROS-responsive polymer materials based on poly(propylene sulfide) [21], selenium-containing copolymers [22], and polythioether ketal [23] have been developed for drug delivery [24, 25]. Unfortunately, these polymers have been limited to clinical applications mainly due to their intrinsic drawbacks such as complex preparation procedures, inevitable drug leakage, poor biocompatibility, low degradability, and considerable cytotoxicity [26, 27]. Compared with ROS-responsive polymer materials, ROS-responsive prodrugs containing oxidation-labile groups are preferred to this end, owing to their advantages of ease of synthesis, higher compatibility, degradability, and reduced off-target toxicity [28, 29]. However, because of the heterogeneity of tumors, the selectivity and efficiency of drug release or activation by endogenous ROS alone in current studies are not significant enough to achieve high anticancer efficacy and minimal side effects.

Nevertheless, a strategy is also available to trigger the drug release by increasing the stimulus concentration at target sites from external forces, such as the intervention of extra light, heat or ultrasonic, which undoubtedly provides a flexible and controllable way to enhance the drug concentration in tumor regions [30–33]. Among all these intervention technologies, photodynamic therapy (PDT) significantly increases the ROS levels by light-activated photosensitizers (PSs) in tumor cells [34, 35]. PDT relies on the local generation of cytotoxic ROS to kill cancer cells, while normal tissues without light irradiation could be free from damage [36]. Thus, a synergistic strategy to realize efficient co-delivery of the PS and ROS-responsive prodrug but decrease the off-target toxicity of chemodrug will have great potential for cancer therapy. ROS generated by PDT could directly kill cancer cells via necrosis or apoptosis, and importantly, trigger the activation of prodrugs for tumor specific chemotherapy. Because its spatial-temporal controllability can be precisely tuned by external triggers, NIR light, this strategy could induce an added inhibitory effect on tumor growth with increased efficiency and low toxicity.

**Scheme 1:**
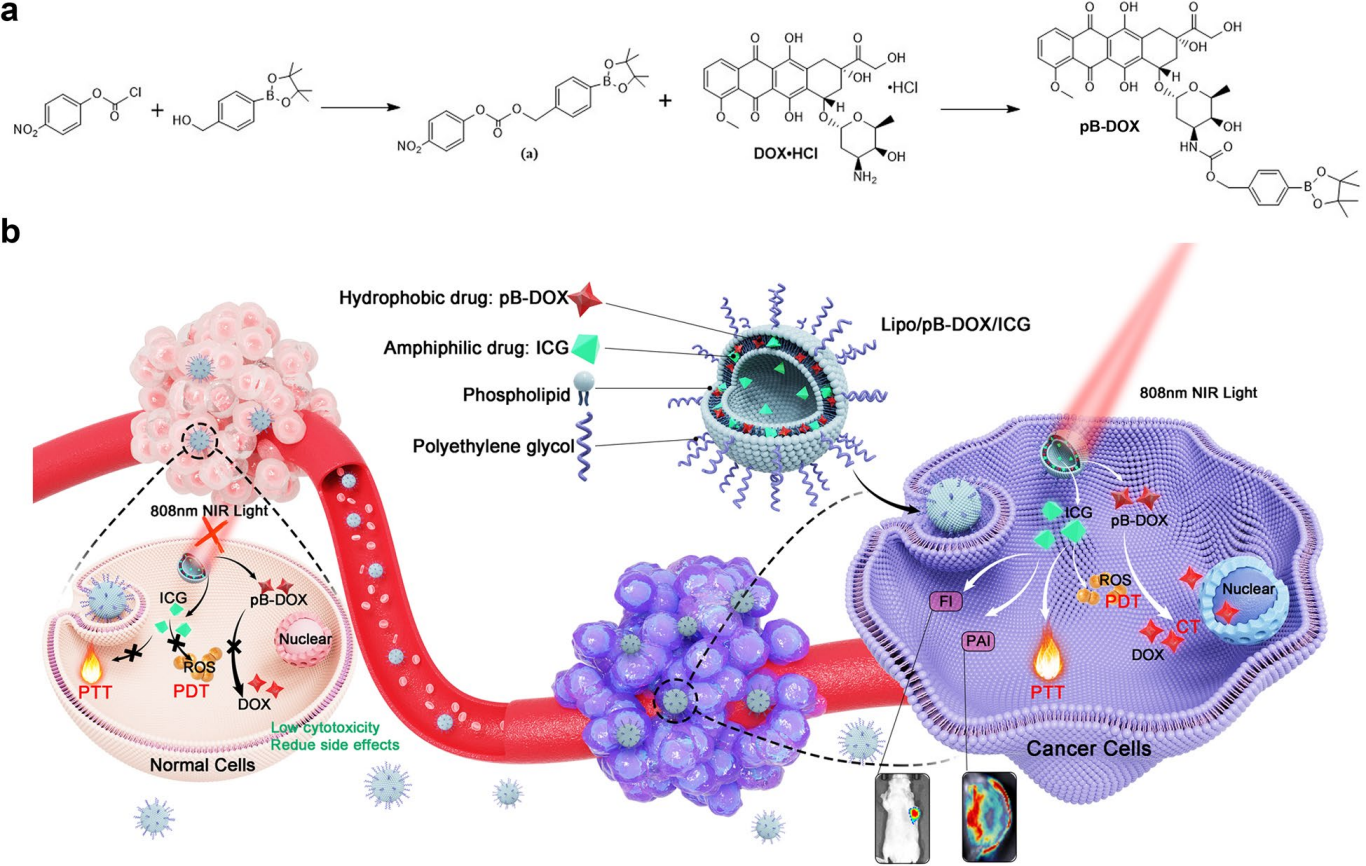
**a** Synthesis of pB-DOX. **b** Schematic illustration of Lipo/pB-DOX/ICG combined PDT with ROS-responsive chemotherapy to synergistically treat cancer cells. (1) The ICG-generated ROS would exert PDT by inducing cell necrosis or apoptosis and selectively cleave the boronate moiety of pB-DOX under 808 nm laser irradiation, triggering DOX release for chemotherapy. (2) ICG could generate localized hyperthermia by absorbing NIR for PTT. (3) Lipo/pB-DOX/ICG offers FI and PAI approaches for visualizing its dynamic distribution within the whole body. (4) With low endogenous ROS levels and without light irradiation, normal tissues could be free from damage

In this study, an ROS-responsive doxorubicin (DOX) prodrug (pB-DOX) was constructed by incorporating a boronate moiety, which reduced its undesired toxicity for normal cells and tissues until ROS activation (Scheme 1a). A novel nanosystem was subsequently developed by simultaneously loading a PS, indocyanine green (ICG), which also acted as a near-infrared (NIR) fluorescence imaging (FI) dye as well as a photoacoustic imaging (PAI) dye, and pB-DOX in polyethylene glycol (PEG) modified liposomes (Lipo/pB-DOX/ICG) (Scheme 1b). Via intravenous injection, Lipo/pB-DOX/ICG was passively targeted in tumor sites, which could be monitored by the FI and PAI of ICG. ICG not only converted the NIR light energy into local hyperthermia for photothermal therapy (PTT), but also produced ROS to participate in PDT and cleave the ROS-sensitive bond of pB-DOX, which persistently restored the DOX activity for chemotherapy. Above all, we developed a novel tool for on-demand drug release at tumor sites, also a promising candidate for controllable and accurate combinatorial tumor therapy.

## Materials and methods

### Materials

1,2-Distearoyl-*sn*-glycero-3-phosphoethanolamine-N-[methoxy(polyethylene glycol)-2000] (DSPE-mPEG2000) and hydrogenated soybean phosphatidylcholine (HSPC) were purchased from Avanti Polar Lipids (Alabaster, AL, USA). Doxorubicin hydrochloride (DOX·HCl) was supplied by Hisun Pharmaceutical (Zhejiang, China). Cholesterol was obtained from Hushi Chemical (Shanghai, China). ICG was supplied by TCI (Tokyo, Japan). 3-(4,5-Dimethylthiazol-2-yl)-2,5-diphenyltetrazolium bromide (MTT reagent) and Hoechst 33,324 were purchased from Sigma-Aldrich (St Louis, MO, USA). Triethylamine (TEA), NaN_3_, and 4-dimethylaminopyridine (DMAP) were obtained from Sinopharm Chemical Reagent (Shanghai, China). Live/Dead viability/cytotoxicity kit was purchased from Invitrogen (Carlsbad, CA, USA). 1,3-Diphenylisobenzofuran (DPBF) and 2′,7′-dichlorofluorescin diacetate (DCFH-DA) were obtained from Beyotime Institute of Biotechnology (Jiangsu, China). The chemicals and solvents were of analytical grade and used as received without further purification.

### Synthesis of pB-DOX and ROS-responsiveness test

The prodrug pB-DOX was synthesized following the literature [11, 37], and the procedure is shown in Scheme 1a.

First, 4-(hydroxymethyl)phenylboronic acid pinacol ester (0.80 g, 3.52 mmol) and DMAP (0.64 g, 5.28 mmol) were dissolved in 20 mL anhydrous dichloromethane (DCM), and the mixture was cooled to 0 °C. Then, a solution of *p*-nitrobenzoyl chloride (1.00 g, 5.00 mmol) in DCM was added dropwise. The mixture was stirred at room temperature for 12 h. The insoluble salt was removed by filtration, and the solution was concentrated by rotary evaporation. The product was purified by column chromatography (silica gel, hexane/ethyl acetate = 3:1 v/v) to afford 1.17 g (86 %) of compound **a** as a white solid.

DOX·HCl (0.40 g, 0.68 mmol), compound **a** (0.40 g, 1.04 mmol), and TEA (143 µL, 2.02 mmol) were dissolved in 8 mL of anhydrous *N*,*N*-dimethylformamide (DMF) and reacted at room temperature in the dark for 10 h. After completion of the reaction, the product was precipitated with diethyl ether and purified by column chromatography (silica gel, DCM/methanol = 9:1 v/v) to provide pB-DOX (0.28 g, 50 %) as a red powder. Its structure and purity were confirmed by ^1^ H NMR analysis (500 MHz, chloroform-d).

Different amounts of H_2_O_2_ were added to pB-DOX in methanol solutions to obtain final H_2_O_2_ concentrations of 0, 1, 100, and 1000 µM. The solutions were incubated at 37 °C. At certain intervals, a 100 µL sample was taken from each group for high-performance liquid chromatography (HPLC) analysis (Agilent, Santa Clara, USA). The mobile phase was acetonitrile/water (6:4 v/v), and the total flow rate was 1.0 mL/min. The fluorescence (FL) at 580 nm with excitation at 480 nm was used to calculate the pB-DOX concentration, and the peak area was integrated and used to calculate the pB-DOX transformation rate.

### Fabrication of Lipo/pB-DOX/ICG

To prepare Lipo/pB-DOX/ICG, a mixture of HSPC, cholesterol, DSPE-mPEG2000, pB-DOX, and ICG with a molar ratio of 12:8:1:1.5:0.75 was dissolved in 5 mL chloroform, and then the mixture was stirred in a rotary evaporator [36, 38]. Then, 5 mL PBS (pH 7.4, 0.01 M) was added, and the mixture was sonicated for 10 min to remove the dried lipid membrane. Then, the suspension was sonicated using an ultrasonic cell disruption system (120 W, 3 s, on, 3 s, off, 3 min) and continuously extruded 20 times using a 200-nm polycarbonate filter. The resulting Lipo/pB-DOX/ICG nanosuspension was stored at 4 °C in the dark until use. Liposomes containing pB-DOX (Lipo/pB-DOX) or ICG (Lipo/ICG) only were prepared in a similar manner.

### Characterization of Lipo/pB-DOX/ICG

The size distribution of Lipo/pB-DOX/ICG, Lipo/pB-DOX, and Lipo/ICG was measured using a Malvern Zetasizer Nano Series instrument (Nano ZS, Malvern Instruments, UK), and their morphology was observed by transmission electron microscopy (TEM, Titan G2-F20, FEI, USA). The aforementioned formulations were monitored by UV-vis-NIR spectroscopy (260-Bio, Thermo Fisher Scientific). After ultrafiltration centrifugation and organic solvent extraction, the encapsulation efficiencies (EE) and loading contents (LC) of pB-DOX and ICG in the liposomes were quantified and calculated using HPLC and UV-vis-NIR spectroscopy, respectively. UV-vis-NIR absorption at 784 nm was used to determine the ICG concentration.

To study the release behavior of pB-DOX and ICG in the liposomes, nanosuspension for each group (1 mL, containing 235 µg of pB-DOX and/or 150 µg of ICG) was sealed in a dialysis bag (MWCO: 3.5 kDa ) and dialyzed in 30 mL PBS with acidic pH (5.5) or 0.2 % Tween 80. At certain intervals, a 100 µL aliquot of the solution outside the bag was withdrawn for HPLC and UV-vis-NIR analyses.

### Singlet oxygen (^1^O_2_) detection and in vitro photothermal effect

DPBF was used to evaluate the in vitro ^1^O_2_ generation capability of Lipo/pB-DOX/ICG, Lipo/ICG, and free ICG. Liposomes (15 µg/mL ICG, 3 mL) were rapidly mixed with fresh DPBF (0.5 mg/mL, 100 µL) and kept in a dark place. Subsequently, the mixture was irradiated using an 808 nm laser (1.0 W/cm^2^). Finally, the absorbance was measured every 20 s at 407 nm wavelength using a UV-vis-NIR spectrophotometer.

To test the photothermal stability of free ICG, Lipo/ICG, and Lipo/pB-DOX/ICG, their changes in the UV-vis-NIR spectra were obtained after the exposure to irradiation with different time periods. The photothermal conversion of various formulations (PBS, free ICG, Lipo/ICG, Lipo/pB-DOX, and Lipo/pB-DOX/ICG) with the same ICG concentration of 12.5 µg/mL was measured under 808 nm laser (1.0 W/cm^2^) irradiation within 5 min using an infrared thermal imaging camera (FLIR E50; Estonia) for real-time thermal imaging. Further, to evaluate the photothermal effect of our optimal formulation, Lipo/pB-DOX/ICG solutions containing different concentrations of ICG (initial ICG concentration: 50 µg/mL) were exposed to an 808 nm laser at 1.0 W/cm^2^ for 5 min, and the temperature changes were recorded using an infrared thermal imaging camera.

### Cell culture

MDA-MB-231 and HEK293 cell lines were obtained from Xiangya Central Experiment Laboratory (Hunan, China). The cells were cultured in Dulbecco’s Modified Eagle Medium containing 10 % fetal bovine serum in a humidified atmosphere containing 5 % CO_2_ at 37 °C [39].

### Cellular uptake

MDA-MB-231 cells (2 × 10^5^ cells per well) were seeded in confocal laser scanning microscopy (CLSM) dishes and cultured overnight. The cells were treated with PBS and different formulations at an identical ICG (3.0 µg/mL) and/or pB-DOX (6 µg/mL) concentration of DOX·HCl (pB-DOX equivalent of 6 µg/mL), Lipo/ICG, Lipo/pB-DOX, and Lipo/pB-DOX/ICG for different time periods. After washing twice with PBS, the cells were successively incubated with Hoechst 33,334 for 15 min. Subsequently, the cells were washed with PBS thrice before observing under a CLSM (Leica, Germany). To analyze the FL intensity quantitatively, flow cytometry was carried out, and the previous operation was performed in the same manner as above. After incubating for different time periods (0, 1, 2, 4, 8, 12, and 24 h), the cells were collected and analyzed using a flow cytometer. To study the effect of ROS on the activity recovery of pB-DOX, the cells were irradiated (808 nm, 1 W/cm^2^) for different time periods after Lipo/pB-DOX/ICG incubation for 2 h, and the FL intensity was determined by inverted FL microscopy and flow cytometry.

### Intracellular ROS detection

DCFH-DA was used to evaluate intracellular ROS generation. MDA-MB-231 cells were seeded with a density of 2 × 10^5^ per well in 12-well plates and treated with 100 mM NaN_3_ [40, 41], a well-known ^1^O_2_ scavenger, to scavenge the original ^1^O_2_ produced by the tumor cells themselves. After incubating for overnight, the medium of each well was replaced with 1 mL of fresh culture medium containing PBS, Lipo/pB-DOX, Lipo/ICG, or Lipo/pB-DOX/ICG (the concentration of ICG is 5.0 µg/mL). The cells were further incubated at 37 °C in 5 % CO_2_ for 2 h. After washing thrice with PBS, the cells were cultured with 1 mL DCFH-DA (25 µM) for 20 min. Subsequently, each well was washed twice with PBS and subjected to 808 nm laser irradiation with 1.0 W/cm^2^ for different time periods. Next, the cells were fixed with 4 % paraformaldehyde solution for 10 min. Finally, the cells were washed thrice with 1 mL PBS. The FL of DCF (λ_ex_ = 495 nm, λ_em_ = 529 nm) was immediately captured by inverted FL microscopy. To analyze the FL intensity quantitatively, flow cytometry (FACSVerse, BD, USA) was used, and the previous operation was performed in the same manner as above.

### In vitro cytotoxicity

The cytotoxicity of combination therapy on MDA-MB-231 cells and security on HEK293 cells under different disposes were assessed using MTT assay. First, MDA-MB-231 cells (1 × 10^4^ cells per well) were incubated in 96-well plates for 24 h. Then, the cells were treated with DOX·HCl, Lipo/ICG + laser, Lipo/pB-DOX, and Lipo/pB-DOX/ICG + laser at different concentrations in media. During incubation, laser irradiation was given at 6 h for 2 min (808 nm, 1.0 W/cm^2^). After 24 h, the cells were further incubated with MTT solution (5 mg/mL, 20 µL) for 4 h. Finally, the obtained formazan crystals were dissolved in DMSO under mild shaking at 37 °C. Immediately, the absorption of each well was measured using a microplate reader (Infinite M200 PRO, TECAN, Austria). The relative cell viabilities without a laser on HEK293 cells were measured in the same manner.

To visualize the phototherapy (including PTT and PDT) efficacy, MDA-MB-231 cells irradiated with the laser were immediately washed thrice with PBS. According to the instructions on the kit, the cells were stained with calcein-acetoxymethyl (AM) for live cells and propidium iodide (PI) for dead cells, followed by inverted FL microscopy (Model IX71 Olympus, Tokyo, Japan). “Image J” software was used to calculate the proportions of live and dead cells. To further evaluate the combination therapy cytotoxicity of liposomes, MDA-MB-231 cells irradiated with/without 808 nm laser (1.0 W/cm^2^) were further incubated for 12 h, and the medium containing drugs was removed and analyzed as before. To evaluate the effect of Lipo/pB-DOX/ICG dosage on toxicity, MDA-MB-231 cells with different doses of Lipo/pB-DOX/ICG (1.0, 5.0, and 15.0 µg/mL pB-DOX) were irradiated with 808 nm laser and further incubated for 12 h, and the medium containing drugs was removed and analyzed as before.

### In vitro FI and PAI

In vitro FI was performed using a Xenogen IVIS Spectrum imaging system (Perkin Elmer, USA) at 745 nm excitation wavelength using an 820 nm filter. The samples were diluted according to the concentration of ICG from 1.0 to 200 µg/mL. PAI experiments were performed using a VEVO LASER PAI system (VEVO 2100, FUJIFILM Visual Sonics, INC, USA). The Lipo/pB-DOX/ICG liposomes were diluted to a series of ICG concentrations from 0 to 200 µg/mL and added to a centrifuge tube for PAI.

### Tumor models

All animal studies were conducted under a protocol approved by the Animal Ethics Committee of Central South University. Breast cancer xenografts were generated by the subcutaneous injection of 1 × 10^6^ MDA-MB-231 cells in 100 µL of serum-free medium onto the right forelimb of BALB/c nude mice (female, 4–5 weeks).

#### In vivo FI and PAI

When the tumor size reached over 200 mm^3^, mice bearing MDA-MB-231 tumors were intravenously injected with free ICG, Lipo/ICG, and Lipo/pB-DOX/ICG (ICG: 3.5 mg/kg). For the *in viv*o FL imaging, an Xenogen IVIS Spectrum imaging system (Perkin Elmer, USA) was used to observe the ICG FL (Ex: 745 nm, Em: 820 nm) in mice body at predetermined time points. Several mice were sacrificed at 48 h for the *ex vivo* imaging of the heart, liver, spleen, lungs, kidneys, and tumor tissues. For the PAI, PA signals of tumor-bearing mice were monitored using a VEVO LASER PAI system at different time points.

#### In vivo antitumor efficiency

When the tumor volumes reached about 150–200 mm^3^, MDA-MB-231 tumor-bearing mice were randomly divided into seven groups (five per group): PBS, PBS + laser, DOX·HCl, Lipo/ICG + laser, Lipo/pB-DOX, Lipo/pB-DOX/ICG, and Lipo/pB-DOX/ICG + laser (DOX equivalent of 3.0 mg/kg, ICG of 3.5 mg/kg). The treatment was carried out by intravenous injection, and the laser groups were irradiated for 24 h after injection at a power intensity of 1 W/cm^2^ for 2 min. The temperature changes in tumors during laser irradiation were monitored using an infrared thermal imaging camera. Tumor volumes and body weights were recorded every 3 days for 21 days. Tumor volume was calculated as follows: tumor volume = length × (width)^2^/2. Tumor growth curves were plotted using the average tumor volume vs. days after the first treatment. All the mice were sacrificed 21 days after the first treatment, and their tumors were resected, weighed, and fixed in formalin for paraffin embedding. Hematoxylin and eosin (H&E) was used to monitor the changes in major organs and tumor tissues after treatment. Inhibition rate of tumor growth (IRT) was calculated as follows: IRT = 100 % × (mean tumor weight of the control group – mean tumor weight of the experimental group) / mean tumor weight of the control group.

### Statistical analysis

Data are expressed as mean ± standard deviation. Statistical significance was evaluated using the Student *t*-test when the groups showed different variances. P values < 0.05 were considered statistically significant (NS, no significance, *p < 0.05, **p < 0.01, ***p < 0.001, ****p < 0.0001).

## Results

### Synthesis and characterization of pB-DOX

**Fig. 1:**
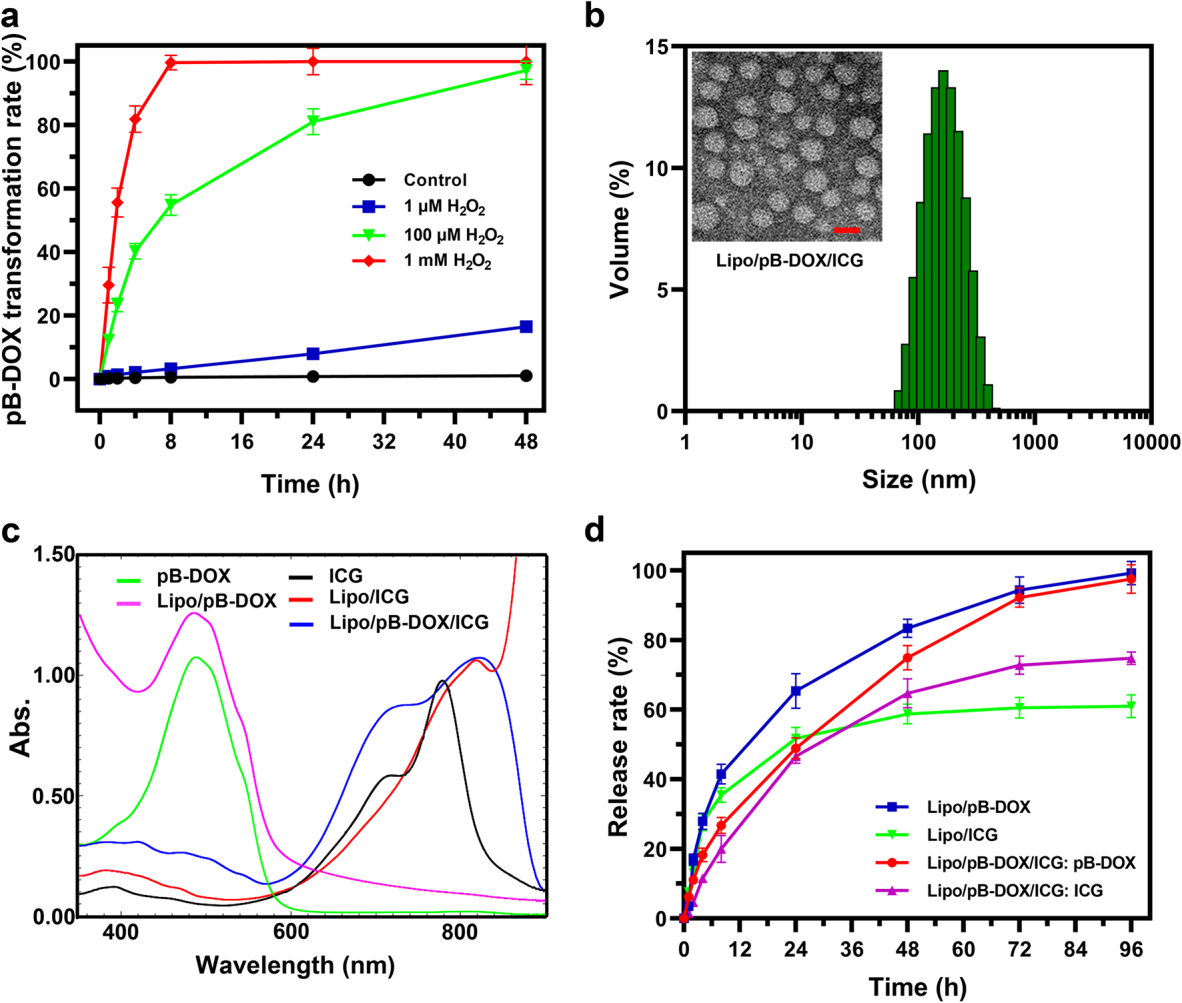
**a** Conversion of pB-DOX to DOX at different concentrations of H_2_O_2_. **b** Size and morphology of Lipo/pB-DOX/ICG measured using a Malvern Zetasizer Nano Series instrument and TEM. Scale bar for TEM is 100 nm. **c** UV-vis-NIR spectra of free ICG and pB-DOX, Lipo/pB-DOX, Lipo/ICG, and Lipo/pB-DOX/ICG. **d** pB-DOX and ICG release from the nanoliposomes in PBS containing 0.2 % Tween 80

The ROS-responsive prodrug pB-DOX was synthesized through a TEA-catalyzed reaction between *p*-nitrobenzoyl-activated 4-(hydroxymethyl) phenylboronic acid pinacol ester and DOX·HCl (Scheme 1a), and its structure and purity were verified by ^1^ H NMR (Additional file 1: Figure S1). To explore the ROS-responsive capability of the prodrug, pB-DOX was incubated with different concentrations of H_2_O_2_, and its activation rates were monitored by measuring the pB-DOX concentrations at different time periods. Figure 1a shows that almost 100 % pB-DOX was transformed into DOX at 4 h when the H_2_O_2_ concentration was above 1 mM. The activation rate at 24 h was up to 81 % in 100 µM H_2_O_2_ solution, while less than 10 % DOX was generated in 1 µM H_2_O_2_ solution within 24 h, and almost no pB-DOX was activated in the absence of H_2_O_2_.

### Synthesis and characterization of Lipo/pB-DOX/ICG

In this multifunctional liposome, ICG, an amphiphilic drug, was wrapped both in the hydrophobic layer and hydrophilic cavity of liposome, and the hydrophobic pB-DOX was encapsulated in the hydrophobic layer. The average hydrodynamic diameter of Lipo/pB-DOX/ICG measured using a Malvern Zetasizer Nano Series instrument was about 170.5 ± 4.1 nm (Fig. 1b and Additional file 1: Table S1), consistent with the TEM results (Fig. 1b). The sizes of Lipo/pB-DOX and Lipo/ICG were 141.4 ± 5.5 nm and 143.5 ± 4.3 nm, respectively. The TEM results show that the three types of liposomes were monodispersed, spherical, and uniform in size (Fig. 1b and Additional file 1: S2).

The characteristic peaks of UV absorption for free pB-DOX and ICG appear at 480 nm and 780 nm, respectively. To verify whether pB-DOX and ICG were successfully encapsulated into the nanoparticles, we characterized the formulations by UV-vis-NIR spectroscopy, and the characteristic peaks of free pB-DOX and/or ICG were also observed in Lipo/pB-DOX, Lipo/ICG, and Lipo/pB-DOX/ICG. Notably, there was a slight red-shift from 780 nm to 815 nm in the absorption spectra of Lipo/ICG and Lipo/pB-DOX/ICG compared to that of free ICG (Fig. 1c).

According to the ultrafiltration centrifugation analysis, the calculated EE values of ICG and pB-DOX in Lipo/pB-DOX/ICG were 55.5 ± 3.2 % and 75.0 ± 2.4 %, respectively; furthermore, the LC values were 3.9 ± 0.6 % and 1.8 ± 0.3 %, respectively (Table S1). The EE and LC values of Lipo/pB-DOX and Lipo/ICG were 58.3 ± 3.2 % and 84.0 ± 2.4 %, and 4.2 ± 0.6 % and 6.0 ± 0.3 %, respectively.

To explore the drug release process, the nanosuspensions were sealed in dialysis bags under PBS solutions with acidic pH (5.5) or 0.2 % Tween 80 to mimic different physiological environments, and the release profiles were recorded by monitoring the drug concentration outside the bag using UV-vis-NIR absorption and HPLC. In PBS solutions (pH = 7.4, 0.01 M) with 0.2 % Tween 80, the release rate of pB-DOX and ICG in Lipo/pB-DOX/ICG reached 97.6 and 74.8 % within 96 h (Fig. 1d). At the same time, the release rates of pB-DOX and ICG in Lipo/pB-DOX and Lipo/ICG reached 99.2 and 61.0 %, respectively. Additionally, the release of pB-DOX and ICG was unsusceptible to acidic pH (5.5) (Additional file 1: Figure S3).

#### In vitro ^1^O_2_detection

**Fig. 2:**
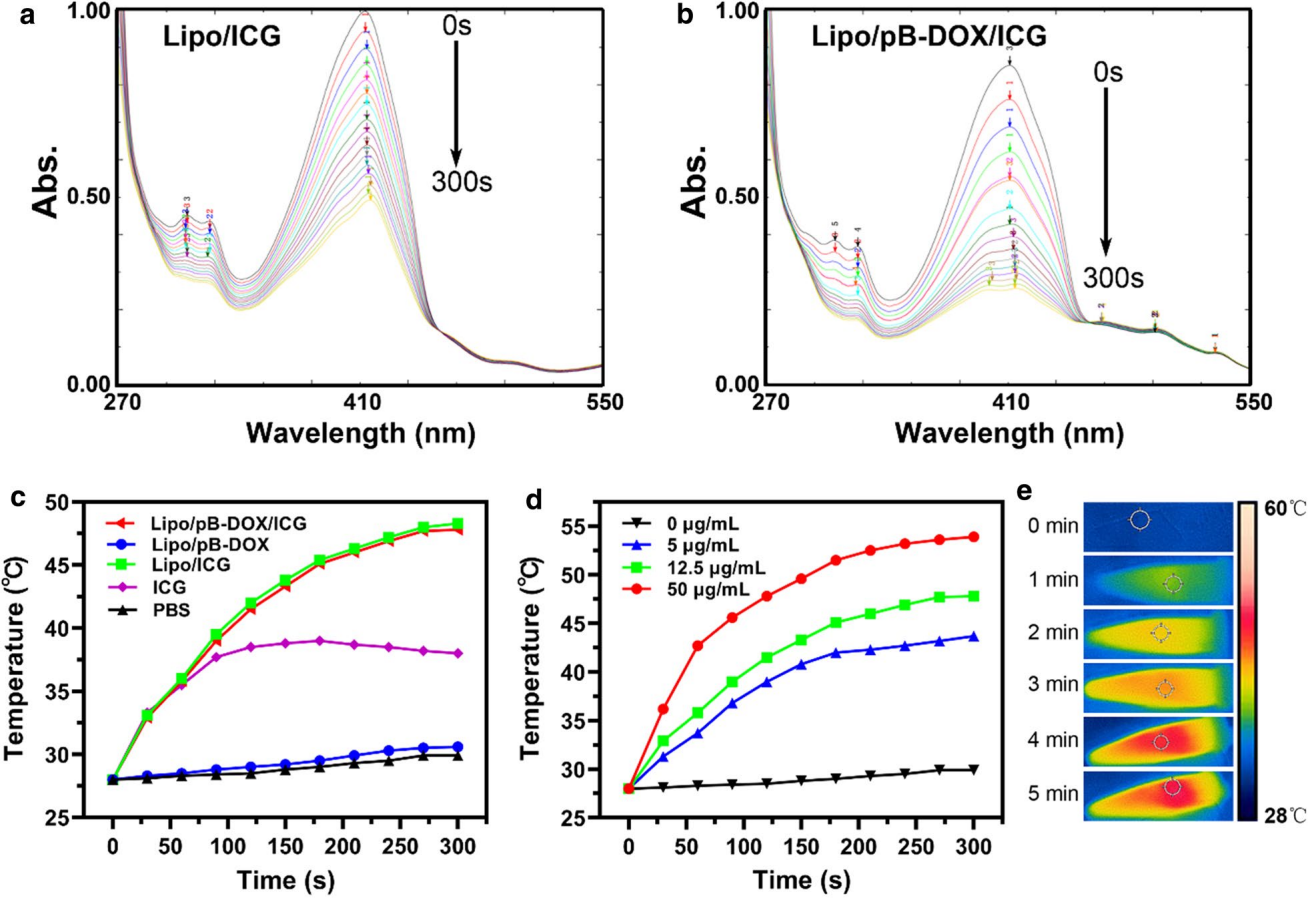
*In vitro* ^1^O_2_ detection of Lipo/ICG **a** and Lipo/pB-DOX/ICG **b** (containing 15 µg/mL ICG) using ^1^O_2_ probe DPBF, whose attenuation rate at 407 nm is directly proportional to the generation rate of ^1^O_2_ under 808 nm laser radiation (1.0 W/cm^2^, 300 s). **c** Temperature rise profiles of PBS, free ICG, Lipo/pB-DOX, Lipo/ICG, and Lipo/pB-DOX/ICG groups, all containing 12.5 µg/mL ICG, and PBS group under 808 nm laser irradiation (1.0 W/cm^2^, 5 min ). **d** Temperature rise profile of Lipo/pB-DOX/ICG with different ICG concentrations (ranging from 0 to 50 µg/mL) under 808 nm laser irradiation (1.0 W/cm^2^, 5 min ). **e** Infrared thermographic maps of Lipo/pB-DOX/ICG (12.5 µg/mL) at different time points under 808 nm laser irradiation (1.0 W/cm^2^)

The ICG-mediated ^1^O_2_ generation capability of liposomes by laser irradiation was detected using a ^1^O_2_ probe DPBF [40]. By observing the UV absorption changes of DPBF at 407 nm, the ability of ^1^O_2_ generation could be evaluated. As shown in Fig. 2a, b, the ^1^O_2_ probe DPBF showed a relatively high degradation rate in the presence of Lipo/ICG or Lipo/pB-DOX/ICG under 808 nm laser (1.0 W/cm^2^). Within 300 s (20 s, 15 times), the absorption of DPBF in the Lipo/ICG and Lipo/pB-DOX/ICG nanosuspension showed 50.1 and 61.3 % decrease, respectively. The absorption of free ICG decreased 78 % under the same condition (Additional file 1: Figure S4).

#### In vitro photothermal evaluation

After irradiating with different time periods, the photothermal stability of free ICG, Lipo/ICG, and Lipo/pB-DOX/ICG was recorded by UV-vis-NIR spectra (Additional file 1: Figure S5). The results show that the absorbance of Lipo/ICG and Lipo/pB-DOX/ICG decreased by 30 and 40 % within 3 min laser illumination, respectively, while free ICG had already lost 88 % of its initial absorbance.

The photothermal effect of the three types of liposomes with the same ICG concentration (12.5 µg/mL) was also monitored. The temperatures of Lipo/pB-DOX/ICG and Lipo/ICG rapidly increased to 47.8 and 48.3 °C within 5 min, whereas the temperatures of free ICG, Lipo/pB-DOX, and PBS (control) increased by only 10.0, 2.6, and 1.9 °C, respectively (Fig. 2c).

To evaluate the photothermal effect of Lipo/pB-DOX/ICG, an infrared thermal imaging camera was used to monitor the temperature changes in Lipo/pB-DOX/ICG nanosuspension with different ICG concentrations under 808 nm laser (1.0 W/cm^2^, 5 min). In the cases of 0, 5, 12.5, and 50 µg/mL of Lipo/pB-DOX/ICG, the temperatures reached up to 29.9, 43.7, 47.8, and 53.9 °C, respectively (Fig. 2d), demonstrating the essential temperature-rise effect and concentration-dependent effect of Lipo/pB-DOX/ICG. The infrared thermal images of Lipo/pB-DOX/ICG (12.5 µg/mL) are also shown in Fig. 2e, indicating the unexceptional PTT effect of the optimal formulation.

### Cellular uptake

**Fig. 3:**
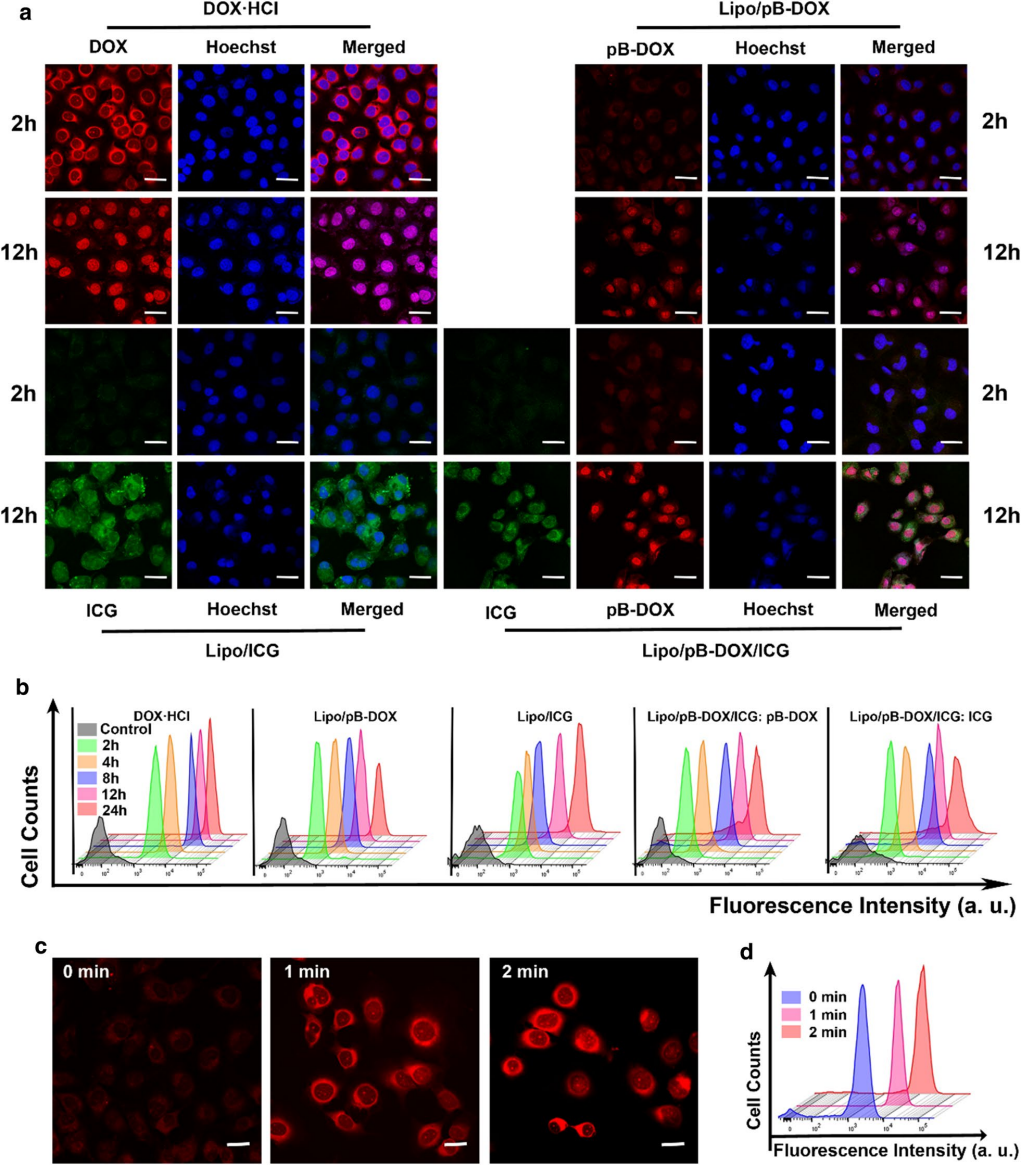
**a** CLSM images of MDA-MB-231 cells incubated with DOX·HCl, Lipo/pB-DOX, Lipo/ICG, and Lipo/pB-DOX/ICG for 2 or 12 h. The scale bar is 50 μm. **b** Flow cytometry analysis of MDA-MB-231 cells incubated with different treatment groups for different incubation periods. **c** CLSM images of MDA-MB-231 cells incubated with Lipo/pB-DOX/ICG and irradiated for different incubation periods. The scale bar is 20 μm. **d** Flow cytometry analysis of MDA-MB-231 cells incubated with Lipo/pB-DOX/ICG and irradiated for different incubation periods. (Ex: 735 nm/Em: 805 nm for ICG; Ex: 505 nm/Em: 550 nm for pB-DOX)

CLSM was further used to observe the subcellular drug distribution and release in cells treated with DOX·HCl, Lipo/ICG, Lipo/pB-DOX, and Lipo/pB-DOX/ICG (Fig. 3). Figure 3a shows that the FL intensity of pB-DOX (or DOX·HCl) was mainly distributed in the cytoplasm after 2 h incubation with DOX·HCl, Lipo/pB-DOX, and Lipo/pB-DOX/ICG. A strong nuclear FL intensity was observed after 12 h incubation. In addition, Lipo/pB-DOX and Lipo/pB-DOX/ICG exhibited lower FL intensity of pB-DOX than DOX·HCl both at 2 and 12 h. The FL intensity of ICG in Lipo/ICG and Lipo/pB-DOX/ICG was also enhanced by prolonging the incubation time. Unlike DOX or pB-DOX distribution, ICG was mainly distributed in the cytoplasm both at 2 and 12 h. Combined with flow cytometry data shown in Fig. 3b, this shows that the FL intensity of both pB-DOX (or DOX) and ICG was directly proportional to the incubation time in a certain time period. After 12 h, the FL intensity did not increase significantly, indicating that the cellular uptake reached saturation.

The FL of DOX, which sharply decreases upon conversion into the prodrug pB-DOX, could be recovered once pB-DOX is transformed into DOX. Therefore, to further confirm that the ROS produced by ICG is sufficient to induce intracellular DOX activation, the cells incubated with Lipo/pB-DOX/ICG for 2 h were irradiated (808 nm, 1 W/cm^2^) for different time periods, and the DOX FL change in cells was monitored. As shown in Fig. 3c, d, the FL intensity of pB-DOX was significantly enhanced after irradiation, and the increase was directly proportional to the irradiation time.

### Intracellular ROS detection

**Fig. 4:**
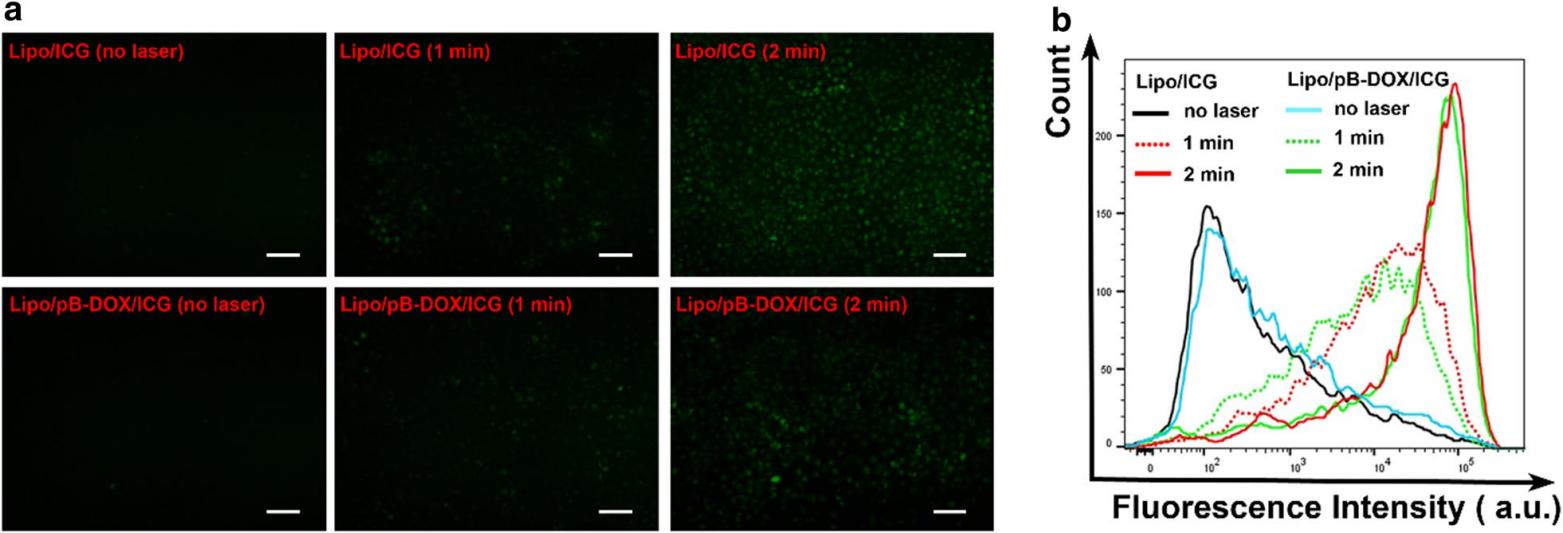
**a** FL images of intracellular ROS generation of Lipo/ICG and Lipo/pB-DOX/ICG with laser (1.0 W/cm^2^) for different time periods (0, 1, and 2 min) inside MDA-MB-231 cells. **b** Flow cytometry analysis of MDA-MB-231 cells after different treatments. (The scale bar is 100 μm. Ex: 488 nm, Em: 525 nm)

Pretreated with NaN_3_ to scavenge endogenous ^1^O_2_ of cancer cells, the intracellular ROS produced by liposomes with laser irradiation was detected using an ROS-sensitive probe DCFH-DA. FL images of MDA-MB-231 cells incubated with PBS, Lipo/pB-DOX, Lipo/ICG, or Lipo/pB-DOX/ICG (the concentration of ICG is 5.0 µg/mL) are shown in Fig. 4a. As shown in the images, Lipo/ICG and Lipo/pB-DOX/ICG showed no FL before irradiation. After laser treatment, both Lipo/ICG and Lipo/pB-DOX/ICG produced green FL, and the FL intensity increased with prolonged irradiation time, consistent with the results of flow cytometry analysis (Fig. 4b). In contrast, PBS and Lipo/pB-DOX groups treated with the same process showed negligible green FL under as long as 2 min irradiation (Additional file 1: Figure S6).

### Cellular cytotoxicity studies

**Fig. 5:**
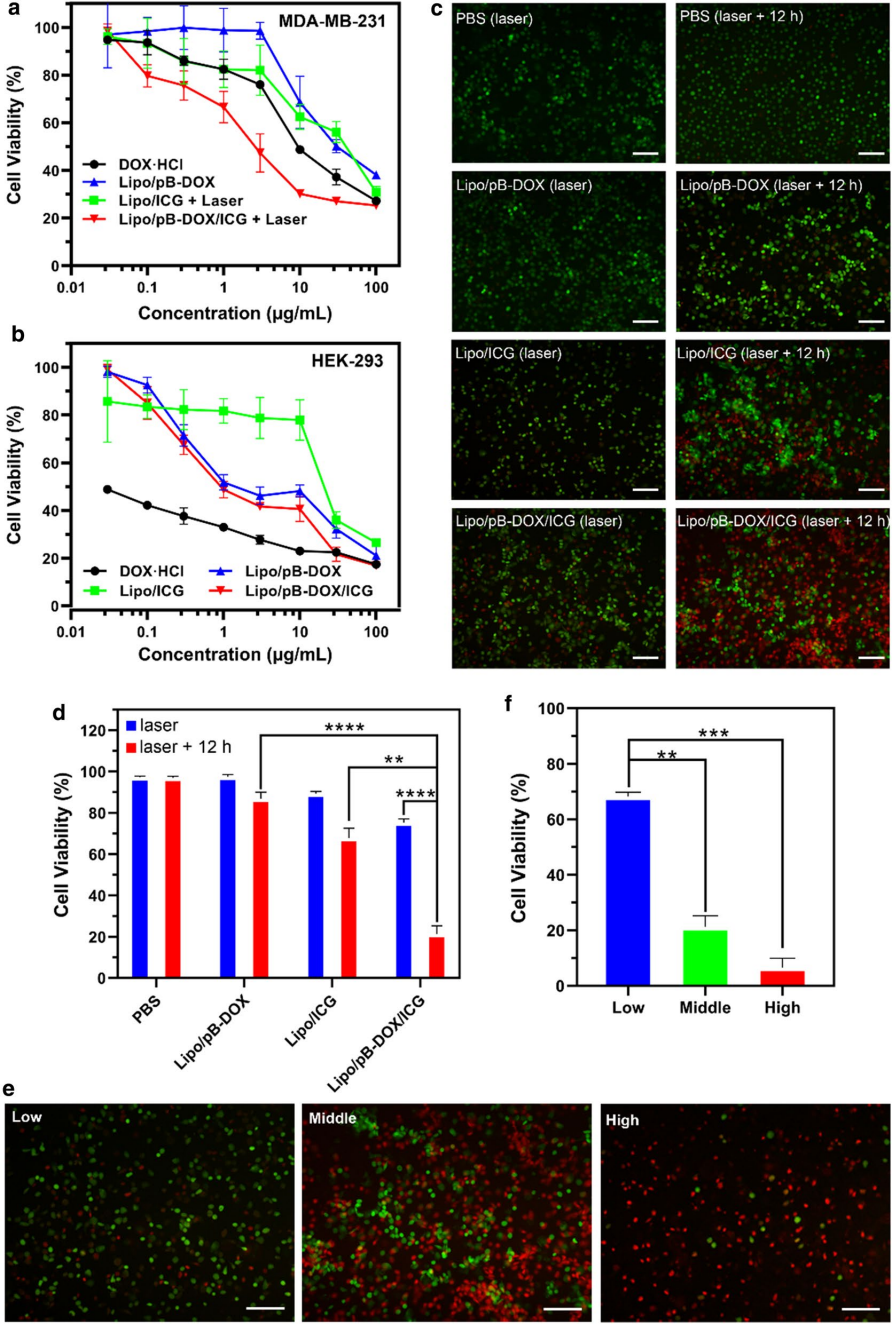
MTT assays of DOX·HCl, Lipo/pB-DOX, Lipo/ICG, and Lipo/pB-DOX/ICG over 24 h in breast cancer cells MDA-MB-231 **a** and normal cells HEK293 **b**. **c** FL images of PI and AM costained MDA-MB-231 cells treated with PBS and different formulations for different incubation periods after irradiation. **d** Cell viabilities of MDA-MB-231 cells treated with PBS and different formulations calculated using “Image J” software. **e** FL images of PI and AM costained MDA-MB-231 cells treated with Lipo/pB-DOX/ICG at different concentrations of pB-DOX (1.0, 5.0, and 15.0 µg/mL). **f** Cell viabilities of MDA-MB-231 cells treated with different drug concentrations of Lipo/pB-DOX/ICG calculated using “Image J” software. (The live cells are stained green, and the dead cells are stained red. The scale bar is 100 μm. NS, no significance, *p < 0.05, **p < 0.01, ***p < 0.001, ****p < 0.0001)

MTT assay was carried out to quantitatively evaluate the cytotoxicity of Lipo/pB-DOX/ICG with different pB-DOX and ICG concentrations (Fig. 5a). MDA-MB-231 cells were treated with DOX·HCl, Lipo/ICG + laser, Lipo/pB-DOX, and Lipo/pB-DOX/ICG + laser. The cell viabilities decreased with increasing concentration of pB-DOX or ICG. The half maximal inhibitory concentration (IC_50_) values of DOX·HCl, Lipo/ICG + laser, Lipo/pB-DOX, and Lipo/pB-DOX/ICG + laser were 14.4, 34.7, 115.4, and 4.2 µg/mL (calculated from the pB-DOX concentration), respectively. The results show that Lipo/pB-DOX is the least toxic liposome, and Lipo/pB-DOX/ICG + laser has the most optimal efficiency to damage cancer cells compared to other groups. On HEK293 cells, the IC_50_ values of DOX·HCl, Lipo/ICG, Lipo/pB-DOX, and Lipo/pB-DOX/ICG were 0.02, 29.5, 3.13, and 2.67 µg/mL (calculated from the pB-DOX concentration), respectively (Fig. 5b). DOX·HCl is 140.5 and 133.5 times more toxic to normal cells than Lipo/pB-DOX and Lipo/pB-DOX/ICG, respectively.

To visualize the phototherapeutic efficacy of Lipo/pB-DOX/ICG, a calcein-AM (green FL, live cells) and PI (red FL, dead cells) costaining study was performed. MDA-MB-231 cells were treated with PBS, Lipo/pB-DOX, Lipo/ICG, and Lipo/pB-DOX/ICG. Under 808 nm laser irradiation (1.0 W/cm^2^) for 2 min, the cell death rates of Lipo/ICG and Lipo/pB-DOX/ICG were 10.6 and 24.8 % (Fig. 5c, d), respectively. To further evaluate the synergistic therapy cytotoxicity of phototherapy and chemotherapy, the cells were further incubated overnight after irradiation. The cell death rates of Lipo/ICG and Lipo/pB-DOX/ICG reached up to 32.2 and 78.2 %, respectively. However, the cell death rates of PBS and Lipo/pB-DOX were only 2.7 and 13.1 % under the same conditions, respectively (Fig. 5c, d).

When the cells were treated with 1.0, 5.0, and 15.0 µg/mL of Lipo/pB-DOX/ICG, irradiated with 808 nm laser, and further incubated for 12 h, the cell death rates at the three concentrations were 31.8 %, 78.2 %, and 93.5 %, respectively (Fig. 5e, f). These results are consistent with the MTT results (Fig. 5a, b) and indicate that the efficiency of combination therapy is also concentration dependent.

#### In vitro FI and PAI

Owing to the inclusion of ICG that acted as a common FL and PA dye [42, 43], the FI and PAI properties of Lipo/pB-DOX/ICG were evaluated. To characterize the optical properties, the FL intensity of nanoliposomes with different ICG concentrations on a well plate was tested. The FL intensity was directly proportional to the concentration of ICG within a low concentration. When the concentration of ICG was over 20 µg/mL, self-quenching was observed (Additional file 1: Figure S7a). Owing to the significant NIR absorbance of nanoliposomes, it was obvious that the PA signal linearly increased within a lower ICG concentration, and enhanced nonlinearly when the concentration was higher than 20 µg/mL, making it a satisfactory PA contrast agent (Additional file 1: Figure S7b).

#### In vivo biodistribution

**Fig. 6:**
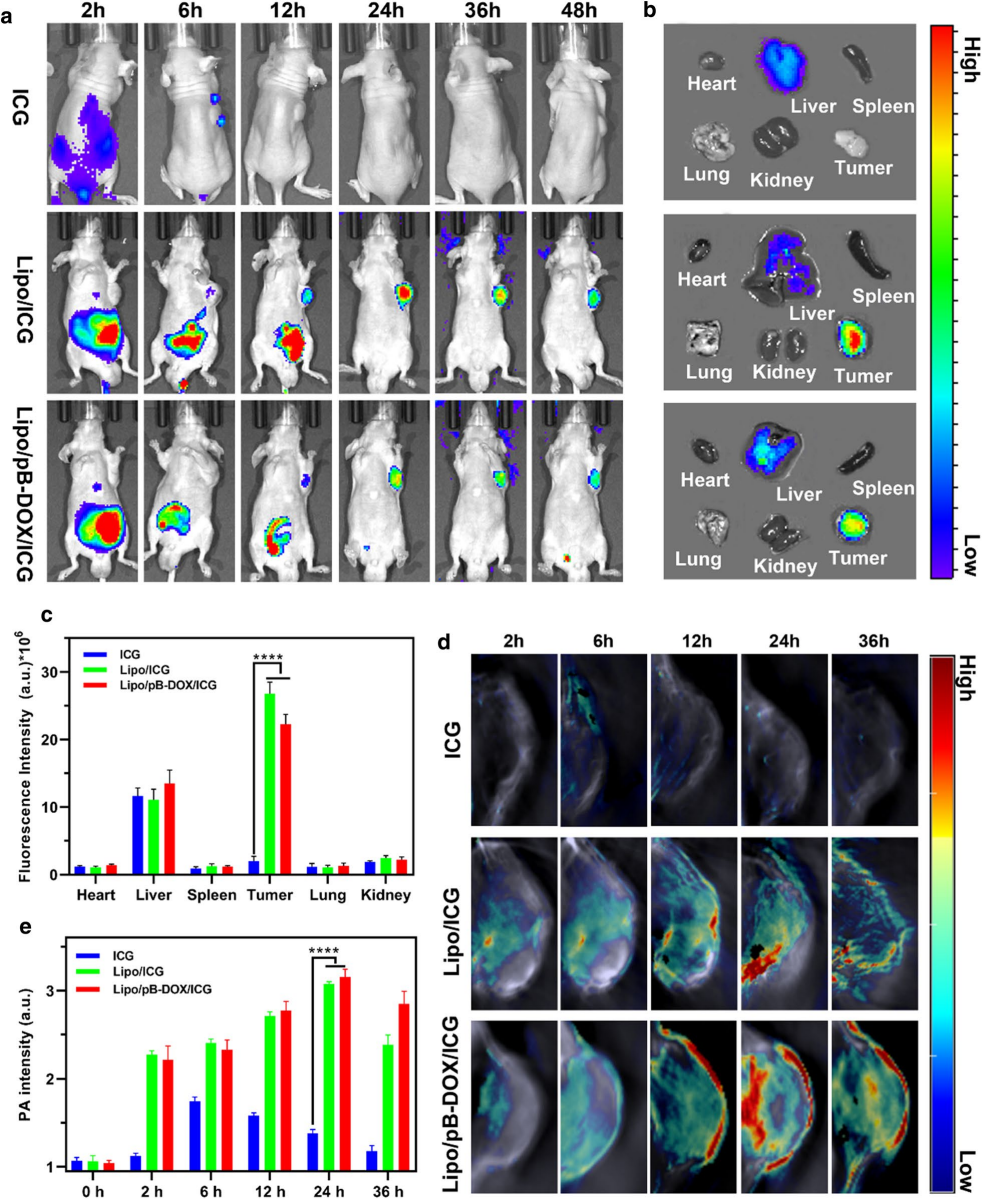
**a** FL images of mice bearing MDA-MB-231 tumors after intravenous injection of free ICG, Lipo/ICG, and Lipo/pB-DOX/ICG at 2, 6, 12, 24, 36, and 48 h. **b** *Ex vivo* FL images of major organs and tumors dissected from mice 48 h post injection of different treatment groups. **c** Semiquantitative biodistribution of free ICG, Lipo/ICG, and Lipo/pB-DOX/ICG in mice determined by the averaged FL intensities of organs and tumor. **d** PA images of tumor sites on MDA-MB-231-tumor-bearing mice post injection of free ICG, Lipo/ICG, and Lipo/pB-DOX/ICG at 2, 6, 12, 24, and 36 h. **e** The corresponding PA signals at the tumor sites of MDA-MB-231-tumor-bearing mice post injection of free ICG, Lipo/ICG, and Lipo/pB-DOX/ICG at different time points. (The data are shown as mean ± SD, n = 3 per group, NS, no significance, *p < 0.05, **p < 0.01, ***p < 0.001, ****p < 0.0001)

ICG has been widely explored as FL and PA contrast agents because of its strong optical absorbance in the NIR region. Both FI and PAI are nonradioactive imaging agents with intrinsic advantages. FI provides a unique approach to visualize the dynamic distribution of nanoparticles throughout the whole body [43]. At the same time, PAI could monitor the accumulation of nanoparticles within the deep tumor region with improved imaging resolution [44]. Nanoliposomes were intravenously injected into nude mice bearing MDA-MB-231 tumors, and the ICG fluorescence was first observed by *in vivo* NIR imaging. Figure 6a shows the quantified FL intensities of ICG at different time points. After 12 h of intravenous injection, considerable FL signals of Lipo/pB-DOX/ICG and Lipo/ICG were observed in the tumor. Then, the nanoliposomes continuously accumulated at the tumor sites and reached the peak at 24 h post injection (Fig. 6a). Owing to the unique long circulation of PEG and EPR effect [45, 46], the mice treated with Lipo/pB-DOX/ICG and Lipo/ICG still exhibited strong FL signals at the tumor sites at 48 h post injection. In contrast, the FL signals in free ICG-treated mice were extensively distributed in the liver, where the FL signals were much stronger than other organs, and no considerable FL signals were observed in the tumor sites.

To confirm tumor accumulation of the nanoliposomes through EPR effect, the tumor bearing mice were sacrificed 48 h post injection, and the tumors and major organs were collected for FI. *Ex vivo* imaging (Fig. 6b) shows bright FL at the tumor regions of mice treated with Lipo/pB-DOX/ICG or Lipo/ICG, and there was no significant difference between the two groups according to the corresponding semiquantitative results (Fig. 6c). However, the FL of free ICG was mainly distributed in the liver, and almost no FL signal occurred in the tumor region. As shown in Fig. 6c, the FL intensity of the tumor region in Lipo/pB-DOX/ICG and Lipo/ICG groups was approximately 13.4-fold and 11.2-fold higher than that in free ICG group, and also much higher than that of other organs within the same groups.

Subsequently, the *in vivo* PAI efficacy of Lipo/pB-DOX/ICG, Lipo/ICG, and free ICG was evaluated using a Vevo LAZR PAI system [47]. After intravenous injection, the PA images of the mice in these groups were obtained at different time periods (Fig. 6d and e). The PAI results confirm that there was no significant difference of distribution between Lipo/pB-DOX/ICG and Lipo/ICG group. The signal intensity in tumor sites gradually increased over time and reached the peak value at 24 h post injection. However, owing to the rapid in vivo clearance of ICG, no obvious PA signal was observed in tumor sites of free ICG group at any time points, and the PAI was about 7.95-fold lower than that of Lipo/pB-DOX/ICG group at 24 h.

#### In vivo combination therapy

**Fig. 7:**
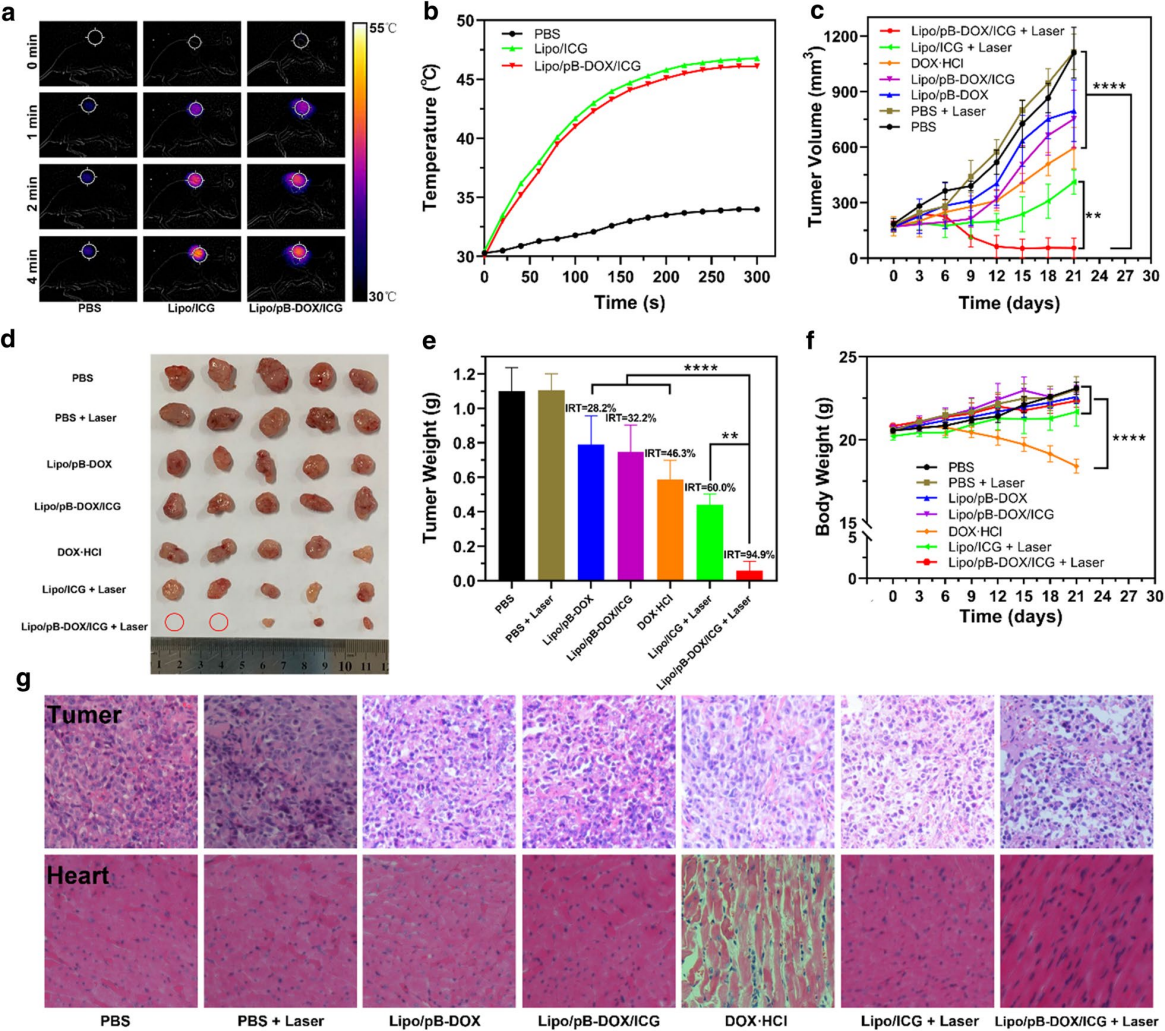
**a** Thermal images of MDA-MB-231-tumor-bearing mice from different treating groups (PBS + laser, Lipo/ICG + laser, and Lipo/pB-DOX/ICG + laser) under 808 nm laser irradiation for different irradiation periods. **b** Temperature change curves at the tumor sites of different treatment groups during 808 nm laser irradiation. **c** Tumor volume curves of different groups after various treatments including: PBS, PBS + laser, DOX·HCl, Lipo/pB-DOX, Lipo/ICG + laser, Lipo/pB-DOX/ICG, and Lipo/pB-DOX/ICG + laser. **d** Photographs of tumor tissues removed from groups treated with different formulations after 21 d. The red circles indicated disappeared tumors. **e** Tumor weights of each group at the end of experiment and IRT. **f** Body weight curves of MDA-MB-231 tumor-bearing mice for each group. **g** Representative histological images of tumor and heart samples from the treated mice. (The data are shown as mean ± SD, n = 5 per group, NS, no significance, *p < 0.05, **p < 0.01, ***p < 0.001, ****p < 0.0001)

Based on excellent imaging ability, photothermal performance of nanoliposomes and the imaging-guided tumor therapy were subsequently investigated *in vivo*. Mice bearing MDA-MB-231 tumors were intravenously injected with Lipo/pB-DOX/ICG or other control formulations, and their tumors were exposed to 808 nm laser irradiation (1.0 W/cm^2^) at 24 h post injection. *In vivo* thermal images were recorded using an infrared thermal imaging camera at different time periods (Fig. 7a). The thermal images showed that the temperature of tumor region in Lipo/pB-DOX/ICG + laser group rapidly reached about 46.1 ℃ (Fig. 7b). Compared to PBS + laser group, Lipo/pB-DOX/ICG + laser group showed prominent ability to increase temperature within a short time, demonstrating that Lipo/pB-DOX/ICG not only could accumulate in tumor sites by passive targeting but also is capable of PTT by laser irradiation to kill tumor cells. The similar temperature curves of Lipo/ICG + laser and Lipo/pB-DOX/ICG + laser groups indicate no significant difference in the photothermal efficiency between them.

To further evaluate the synergistic therapeutic effect, the changes in tumor volumes and mice body weights were recorded every three days after the treatment. Compared with PBS and PBS + laser groups, growth inhibition was observed in all drug-treated groups, and the anticancer activity of DOX·HCl, Lipo/pB-DOX, and Lipo/pB-DOX/ICG groups was very limited because of different deficiencies. The tumor volumes of Lipo/pB-DOX/ICG + laser group showed reduction, indicating the best suppress tumor effect (Fig. 7c). The visualized photograph of tumor tissues treated with different formulations and conditions is shown in Fig. 7d, and the results were consistent with the relative tumor volume change curves (Fig. 7c). The IRT for Lipo/pB-DOX/ICG + laser group was 94.9 %, much higher than 28.2 % for Lipo/pB-DOX group, 32.2 % for Lipo/pB-DOX/ICG group, 46.3 % for DOX·HCl group, and 60.0 % for Lipo/ICG + laser group (Fig. 7e). Beyond that, mice body weight change curves (Fig. 7f) were also used to explore the therapeutic efficacy and toxicity. Compared to obvious changes and loss of body weight in DOX·HCl group, Lipo/pB-DOX/ICG + laser group showed a steady increase of body weight.

To further evaluate the *in vivo* toxicity and antitumor efficacy, a subsequent histological study was carried out by staining tumor tissue and major organs with H&E (Fig. 7g and Additional file 1: S8). Compared with the regularly and tightly packed spherical tumor cells of the PBS group, the tumor cells of Lipo/pB-DOX/ICG + laser group were swollen and exhibited significantly decreased cellularity, severe vacuolization, and nucleus shrinkage, which are typical apoptotic and necrotic characteristics. Vacuolization appeared in the tumor tissues of DOX·HCl and Lipo/ICG + laser groups with a much lesser extent than that of Lipo/pB-DOX/ICG + laser group. The excellent *in vivo* therapeutic effect of Lipo/pB-DOX/ICG + laser against MDA-MB-231 tumors well correlates to the *in vitro* cytotoxicity data (Fig. 5) and proves that the combination (PTT, PDT, and chemotherapy) of pB-DOX and ICG has superior synergistic effect. Because cardiotoxicity is the most prominent defect of DOX [48, 49], the heart tissue sections were stained with H&E for histological study. As shown in Fig. 7g, the cardiac muscle fibers in the other six groups were normal, while DOX·HCl group showed apparent necrosis of myocardial cells, suggesting that Lipo/pB-DOX/ICG has excellent selectivity to circumvent the severe cardiotoxicity of DOX. No treatment-induced adverse effects were observed on the major organs (heart, liver, spleen, lung, and kidney) (Additional file 1: Figure S8).

## Discussion

Owing to the in-depth study of cancer pathology and the rapid development of nanomaterials, specific DDSs for on-demand drug release at tumor sites have become a promising way to controllably and accurately cure cancers [6–10]. A series of ROS-responsive polymer materials and prodrugs containing oxidation-labile groups, such as polypropylene sulfide, selenium group, and polythioether ketal, have offered a novel strategy for cancer treatment [21–23]. For example, Xu et al. reported polyprodrug nanoparticles (denoted as iRGD-NPs), where mitoxantrone was copolymerized with an ROS-cleavable thioketal linker and decorated with iRGD-conjugated DSPE-PEG_3K_ [50]. With specific recognition of iRGD, iRGD-NPs showed a much higher uptake than the nanoparticles without iRGD. However, the complex preparation procedures and inefficient drug release by ROS in this study will undoubtedly reduce its anticancer efficacy and hinder its clinical application. To achieve efficient drug release, Chen et al. constructed a ROS and GSH dual-responsive nanoparticulate DDS (denoted as PTX-TKNs) for paclitaxel delivery [16]. PTX-TKNs could respond to the extracellular ROS and intracellular GSH, achieving a programmable release of paclitaxel at tumor sites. Unfortunately, the high intracellular concentration of GSH in normal cells will lead to inevitable drug leakage, resulting in considerable systemic toxicity.

Herein, an ROS-responsive prodrug pB-DOX was constructed by incorporating a boronate moiety in DOX, thus reducing its undesired toxicity for normal cells and tissues until ROS activation (Scheme 1a). The structure and purity of pB-DOX were verified by ^1^ H NMR (Additional file 1: Figure S1). The results of ROS-responsive capability show that almost 100 % pB-DOX was transformed into DOX at 4 h when the H_2_O_2_ concentration was more than 1 mM (Fig. 1a). The activation rate at 24 h was up to 81 % in 100 µM H_2_O_2_ solution, while less than 10 % DOX was generated in 1 µM H_2_O_2_ solution within 24 h. Almost no pB-DOX was activated in the absence of H_2_O_2_. The intracellular ROS concentration in normal cells is about 20 × 10^− 9^ M, and 1 µM was regarded as the upper limit for the healthy physiological range [20, 51]. Because of the low activation rate at 1 µM, a negligible amount of DOX would be generated in normal cells, thus avoiding the side effects of anticancer drugs. In contrast, the high endogenous ROS level in cancer cells [52] together with ROS generated by the coloaded ICG would achieve a high ROS-responsive capability of pB-DOX.

We subsequently constructed a combination therapy nanosystem by coencapsulating ICG and pB-DOX in PEG-modified liposomes. The particle size of Lipo/pB-DOX/ICG was about 170.5 nm as determined by a Malvern Zetasizer Nano Series instrument and TEM, along with a monodispersed, spherical, and uniform size distribution (Fig. 1b and Additional file 1: Table S1). Besides, nanoliposomes showed the characteristic absorption peaks of both pB-DOX and ICG at about 480 nm and 780 nm, respectively, verifying that pB-DOX and ICG were successfully encapsulated into the nanoparticles (Fig. 1c). The slight red shift from 780 nm to 815 nm in the absorption spectra of Lipo/ICG and Lipo/pB-DOX/ICG can be ascribed to the close-packed ICG in the nanoliposomes or the aggregation of ICG induced by the addition of pB-DOX, which absorbed at a longer wavelength compared to a single ICG molecule [53, 54]. The calculated EE values of ICG and pB-DOX in Lipo/pB-DOX/ICG were about 55.5 and 75.0 %, respectively; the LC values were 3.9 and 1.8 %, respectively (Table S1). In PBS solutions (pH = 7.4, 0.01 M) with 0.2 % Tween 80, pB-DOX and ICG in Lipo/pB-DOX/ICG showed sustained release profiles (Fig. 1d). Additionally, the release of pB-DOX and ICG was unsusceptible to acidic solutions (pH = 5.5) (Additional file 1: Figure S3), thus preventing undesired drug release in cells from the acidic pH in the endosomes and lysosomes and significantly reducing the undesired cytotoxicity in normal cells. Under 808 nm laser irradiation (1.0 W/cm^2^), the ^1^O_2_ probe DPBF showed a relatively high degradation rate in the presence of Lipo/pB-DOX/ICG (Fig. 2b). Because the attenuation rate of DPBF is directly proportional to the generation rate of ^1^O_2_, the results indicate that Lipo/pB-DOX/ICG produced a large amount of ROS, thus playing excellent roles in PDT and the subsequent activation of pB-DOX. Within 3 min laser illumination, the decrease in the absorbance of Lipo/pB-DOX/ICG (40 %) was lower than that of free ICG (88 %) (Additional file 1: Figure S5). The improved photostability of ICG in Lipo/pB-DOX/ICG can be attributed to the protective effect of nanoliposomes by isolating the entrapped ICG from the surrounding environment and reducing the water-induced transformations [55]. Such an improved photostability of nanoliposomes is important for *in vivo* applications. ICG acts as not only a good PS but also an excellent PTT agent, and the temperature rise of Lipo/pB-DOX/ICG was proportional to the illumination time and ICG concentration within a certain range (Fig. 2c–e).

MDA-MB-231 cells exhibited an efficient cell uptake of Lipo/pB-DOX/ICG. A strong nuclear FL of pB-DOX in Lipo/pB-DOX/ICG group was observed after 12 h incubation, while ICG was mainly distributed in the cytoplasm both at 2 and 12 h (Fig. 3a, b). Because DOX acts on DNA and topoisomerase II, both of which are located in the nucleus, DOX accumulation in the nucleus is crucial to induce apoptosis [56]. Unlike DOX or pB-DOX, the distribution of ICG in nucleation or not did not affect its therapeutic effect. In addition, Lipo/pB-DOX/ICG exhibited a lower pB-DOX FL intensity than DOX·HCl both at 2 and 12 h. This is because the FL intensity of pB-DOX is weaker than that of DOX·HCl, and pB-DOX was insufficiently activated in the cell. After incubating for 2 h, the pB-DOX FL intensity in Lipo/pB-DOX/ICG group was significantly enhanced by 808 nm laser irradiation (Fig. 3c, d), and the increase was directly proportional to the irradiation time. The results indicate that the ROS produced by ICG with external irradiation could transform pB-DOX into DOX. Pretreated with NaN_3_ to scavenge endogenous ^1^O_2_ of cancer cells, Lipo/pB-DOX/ICG produced an additional high level of ROS inside tumor cells by laser irradiation (Fig. 4), thus playing an indispensable role in PDT and subsequent initiation of chemotherapy. Under the ROS concentration of tumor microenvironment, the amount of DOX produced by the activation of pB-DOX on MDA-MB-231 cells was lesser (Fig. 5a). Hence, Lipo/pB-DOX was far less toxic than DOX·HCl. Under laser irradiation, Lipo/pB-DOX/ICG not only converted NIR light energy into local hyperthermia for PTT, but also produced an additional high level of ROS to participate in PDT, indicating that the nanoparticles have certain phototherapeutic effect (Fig. 5c, d). With further incubation overnight, pB-DOX was gradually converted to DOX (Fig. 3c, d) by ROS and entered the nucleus for chemotherapy. Therefore, the synergistic therapy efficacy of phototherapy and chemotherapy is far superior to that of phototherapy alone (Fig. 5c–f). On HEK293 cells, DOX·HCl was 140.5 and 133.5 times more toxic to normal cells than Lipo/pB-DOX and Lipo/pB-DOX/ICG, respectively (Fig. 5b), indicating that the design of prodrug in this study dramatically reduces the DOX toxicity on normal cells. The results of cellular cytotoxicity studies show the biosafety of prodrug design and excellent therapy effect of PTT, PDT, and chemotherapy.

After intravenous injection, the signal intensity in tumor sites gradually increased over time and reached the peak value at 24 h post injection. which could be monitored by the FI and PAI of ICG. The FL intensity and PA signal of tumor region in Lipo/pB-DOX/ICG group were much higher than those in free ICG group and other organs within the same group (Fig. 6), indicating that Lipo/pB-DOX/ICG effectively accumulated at the tumor sites by passive targeting. In contrast, the FL signals in free ICG-treated mice were extensively distributed in the liver and negligible in the tumor sites due to tight binding by plasma proteins, lack of target specificity, concentration-dependent self-aggregation, and rapid clearance of free ICG [57–59]. Thanks to the excellent accumulation and external laser irradiation in tumor regions, the combined PTT, PDT, and chemotherapy were realized, specifically in the irradiation sites, and the tumors of Lipo/pB-DOX/ICG + laser group almost disappeared with the highest IRT (94.9 %) (Fig. 7c–e). Furthermore, the mice body weight change curves and histological study demonstrated negligible cardiotoxicity and no treatment-induced side effects of the therapeutic process (Fig. 7f, g).

In general, compared with the recently reported ROS-responsive DDSs, Lipo/pB-DOX/ICG exhibited several advantages, such as on-demand drug release, accurate combinatorial therapy, phototheranostics, and great biosafety for clinical applications. Thus, this ROS-responsive liposome (Lipo/pB-DOX/ICG) developed in this study is a promising candidate for safe and effective tumor therapy.

## Conclusions

In this study, an ROS-responsive nanosystem (Lipo/pB-DOX/ICG) was formed by simultaneously loading the ICG and an ROS-responsive prodrug pB-DOX in PEG-modified liposomes. Notably, Lipo/pB-DOX/ICG displays NIR laser-triggered controllable chemotherapy and phototherapy. Excited by NIR light, Lipo/pB-DOX/ICG not only converted NIR light energy into local hyperthermia for PTT, but also produced large amounts of ROS to achieve PDT, which also triggered the activation of pB-DOX for chemotherapy. Owing to the phototherapy and chemotherapy synergistic effect, Lipo/pB-DOX/ICG displayed a remarkably improved cytotoxicity in MDA-MB-231 cells, with an IC_50_ value of only 29.2 % of that for DOX·HCl. More importantly, it is nontoxic or less toxic during the systemic circulation and in nontumor tissues along with negligible inherent ROS, and DOX·HCl is 133.5 times more toxic to normal cells than Lipo/pB-DOX/ICG. After intravenous injection, the nanoliposomes continuously accumulated at the tumor sites and reached the peak at 24 h post injection, as confirmed by FI and PAI. Lipo/pB-DOX/ICG showed the best behavior to suppress tumor growth through synergistic therapy, which was far superior to the theranostic outcome of Lipo/ICG or DOX·HCl alone. Meanwhile, the design of pB-DOX circumvented the severe cardiotoxicity of DOX, and no significant adverse effects were observed on the major organs. All efforts have reduced the side effects of encapsulated anticancer drugs and substantially enhanced the therapeutic efficacy. In conclusion, Lipo/pB-DOX/ICG is a novel tool for on-demand drug release and achieves superior antitumor efficacy by controllable and accurate combination therapy.

## Supplementary Information

**Additional file 1:** Additional tables and figures.

## Data Availability

All data generated or analyzed during this study are included in this published article and its supplementary information files.

## Abbreviations

AM: Acetoxymethyl
CLSM: Confocal laser scanning microscopy
DCFH-DA: 2′,7′-dichlorofluorescin diacetate
DDSs: Drug delivery systems
DMAP: 4-dimethylaminopyridine
DMF: *N*,*N*-dimethylformamide
DOX: Doxorubicin
DOX·HCl: Doxorubicin hydrochloride
DPBF: 1,3-diphenylisobenzofuran
DSPE-mPEG2000: 1,2-distearoyl-*sn*-glycero-3-phosphoethanolamine-N-[methoxy(polyethylene glycol)-2000]
EE: Encapsulation efficiencies
EPR: Enhanced permeation and retention
FDA: Food and Drug Administration
FI: Fluorescence imaging
FL: Fluorescence
GSH: Glutathione
H&E: Hematoxylin and eosin
HEK293: Human embryonic kidney cell line
HPLC: High-performance liquid chromatography
HSPC: Hydrogenated soybean phosphatidylcholine
H_2_O_2_: Hydrogen peroxide
ICG: Indocyanine green
IC_50_: Half maximal inhibitory concentration
IRT: Inhibition rate of tumor growth
LC: Loading contents
Lipo/ICG: Liposomes containing ICG
Lipo/pB-DOX: Liposomes containing pB-DOX
Lipo/pB-DOX/ICG: Liposomes containing pB-DOX and ICG
MDA-MB-231: Human breast cancer cell line
NIR: Near-infrared
PAI: Photoacoustic imaging
pB-DOX: Doxorubicin prodrug
PDT: Photodynamic therapy
PEG: Polyethylene glycol
PI: Propidium iodide
PSs: Photosensitizers
PTT: Photothermal therapy
ROS: Reactive oxygen species
TEA: Triethylamine
TEM: Transmission electron microscopy
