# Supplementary material for: ROS-responsive liposomes with NIR light-triggered doxorubicin release for combinatorial therapy of breast cancer
Source: J Nanobiotechnology. 2021 May 11;19:134. doi: 10.1186/s12951-021-00877-6 (PMC8111982; doi:10.1186/s12951-021-00877-6)
Supplement: Supplementary file 1 — Additional file 1: Additional tables and figures. [file 12951_2021_877_MOESM1_ESM.docx]

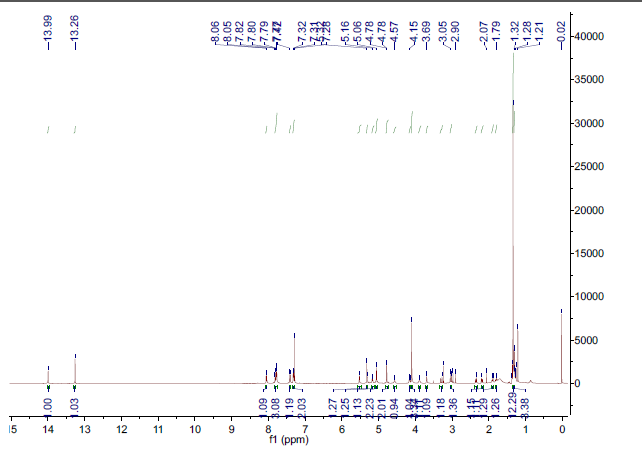


**Figure S1** ^1^H NMR spectrum of pB-DOX in CDCl_3_. δppm: 13.99 (s, 1H), 13.26 (s, 1H), 8.05 (d, J = 7.7 Hz, 1H), 7.81 (d, J = 8.2 Hz, 1H), 7.78 (d, J = 8.0 Hz, 2H), 7.41 (d, J = 8.4 Hz, 1H), 7.32 (d, J = 7.6 Hz, 2H), 5.52 (d, J = 3.9 Hz, 1H), 5.31 (s, 1H), 5.17 (d, J = 8.4 Hz, 1H), 5.06 (s, 2H), 4.78 (d, J = 2.2 Hz, 2H), 4.19–4.13 (m, 1H), 4.10 (s, 3H), 3.94–3.85 (m, 1H), 3.69 (s, 1H), 3.27–3.32 (m, 1H), 2.90–2.98 (m, 1H), 2.35 (dd, J = 14.7, 2.3 Hz, 1H), 2.19 (dd, J = 14.7, 4.0 Hz, 1H), 1.92–1.88 (m, 1H), 1.81 (dd, J = 13.2, 4.1 Hz, 1H), 1.34 (s, 12H), 1.31 (d, J = 6.6 Hz, 3H).

**Table S1** Characteristics of Lipo/pB-DOX, Lipo/ICG, and Lipo/pB-DOX/ICG.

|  | Diameter (nm) | EE_(pB-DOX)_  % | EE_(ICG)_  % | LC_(pB-DOX)_  % | LC_(ICG)_  % |
| --- | --- | --- | --- | --- | --- |
| Lipo/pB-DOX/ICG | 170.5 ± 4.1 | 55.5 ± 3.2 | 75.0 ± 2.4 | 3.9 ± 0.6 | 1.8 ± 0.3 |
| Lipo/pB-DOX | 141.4 ± 5.5 | 58.3 ± 3.2 | - | 4.2 ± 0.6 | - |
| Lipo/ICG | 143.5 ± 4.3 | - | 84.0 ± 2.4 | - | 6.0 ± 0.3 |


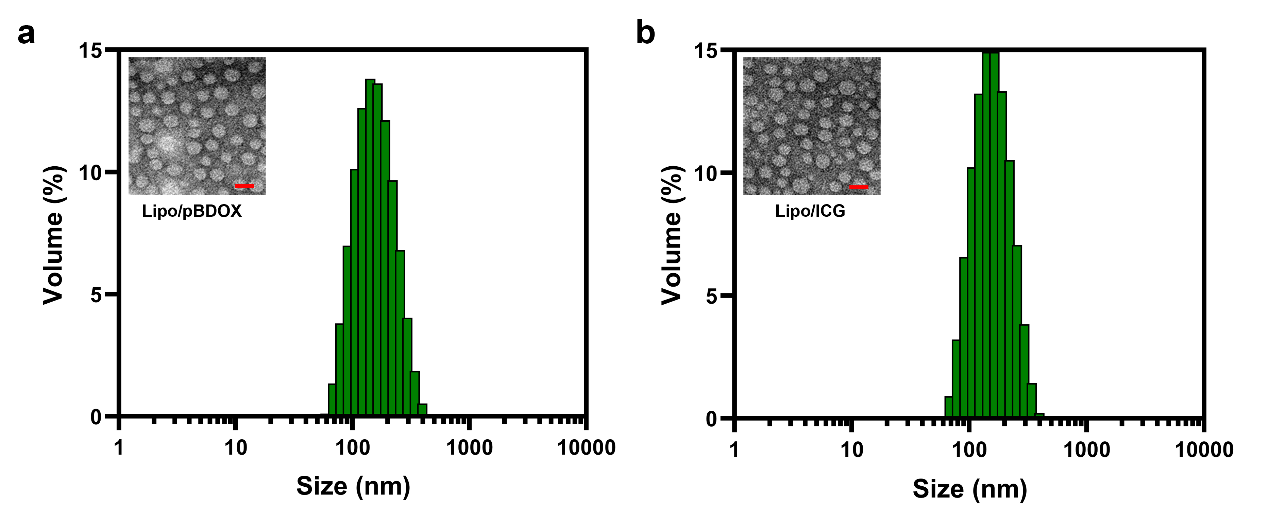


**Figure S2** Size and morphology of Lipo/pB-DOX **a** and Lipo/ICG **b** measured using a Malvern Zetasizer Nano Series instrument and TEM. Scale bar for TEM is 100 nm.


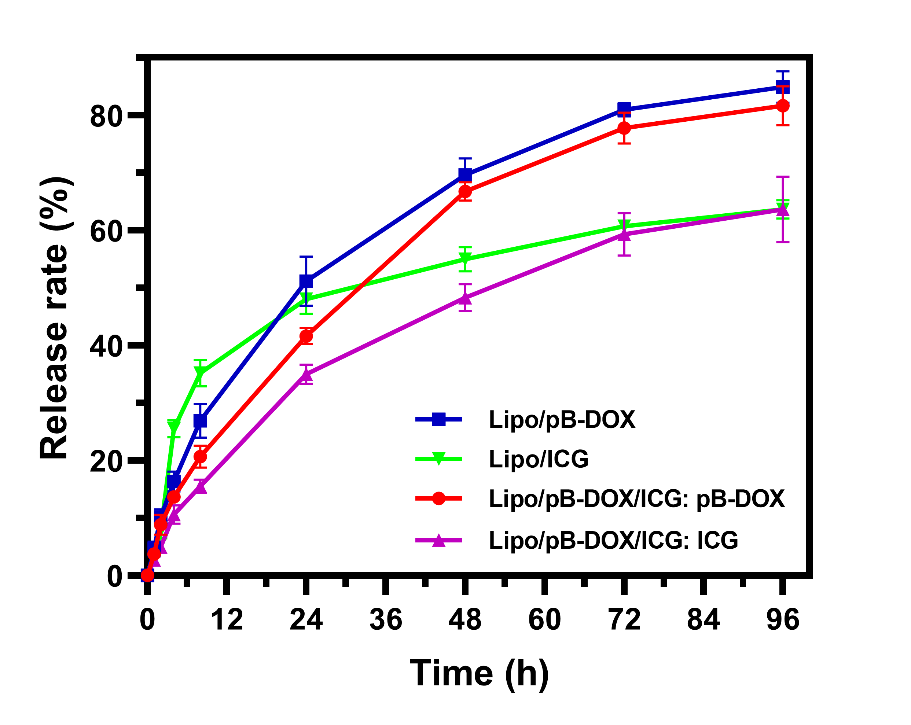


**Figure S3** pB-DOX and ICG release from the nanoliposomes in PBS with acidic pH (5.5).


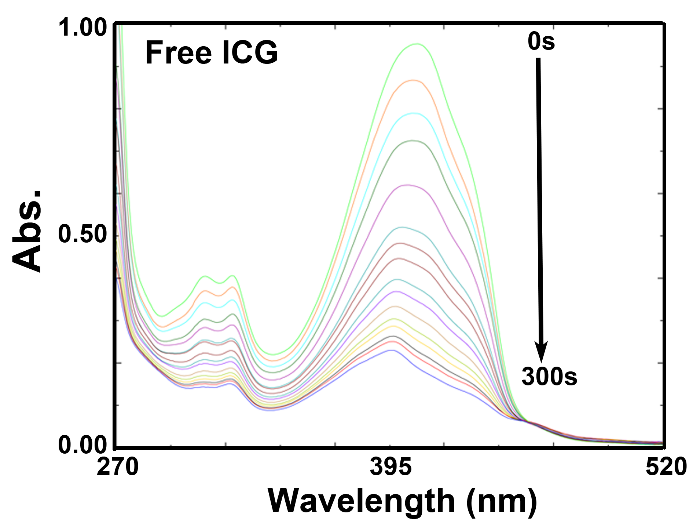


**Figure S4** The degradation of DPBF over time at 407 nm under continuous 808 nm laser radiation (1.0 W/cm^2^, 300 s) for free ICG (15 μg/mL).


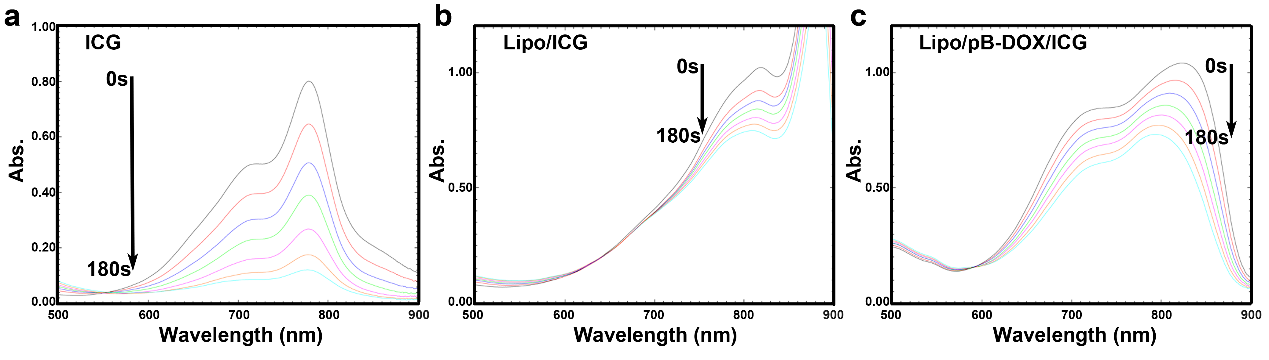


**Figure S5** UV-vise-NIR spectra of free ICG **a**, Lipo/ICG **b**, and Lipo/pB-DOX/ICG **c** at different time points under 808 nm laser irradiation (1.0 W/cm^2^, 3 min).


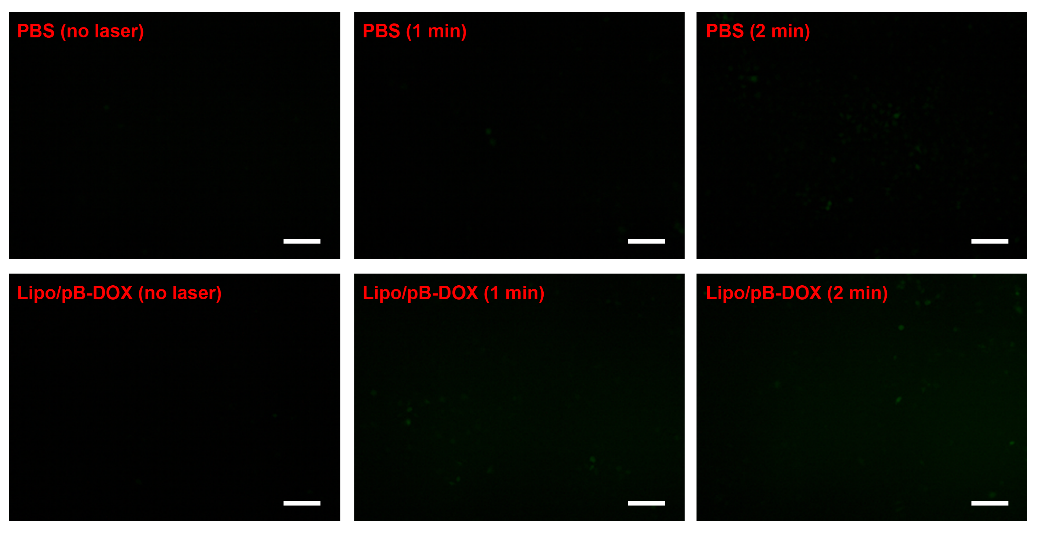


**Figure S6** Fluorescence images of intracellular ROS generation of PBS and Lipo/pB-DOX with laser (1.0 W/cm^2^) for different time periods (0, 1 and 2 min) inside MDA-MB-231 cells. The scale bar is 100 μm.


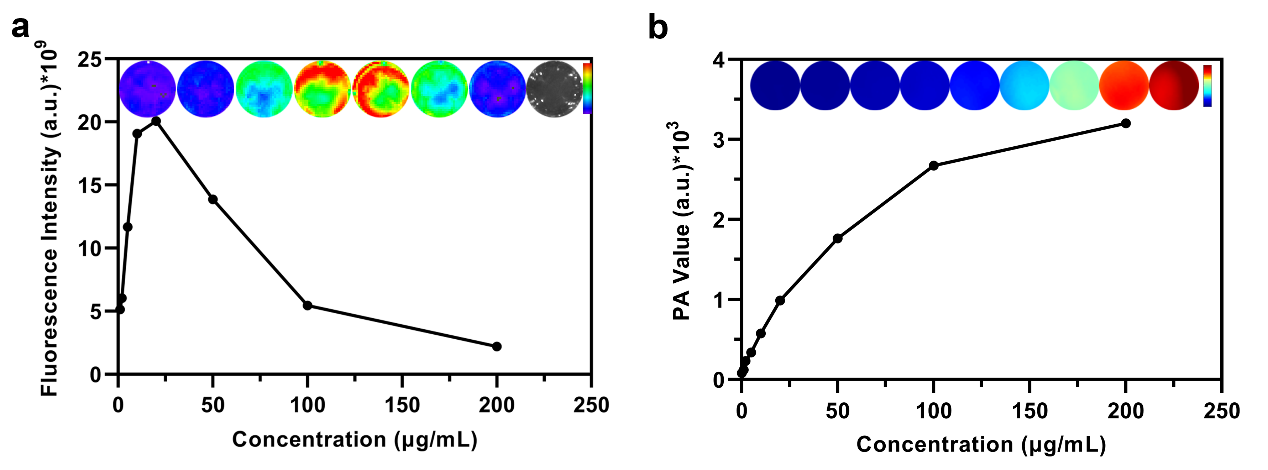


**Figure S7** **a** FL intensities and FL images of Lipo/pB-DOX/ICG with different concentrations of ICG. **b** PA signals and PA images of Lipo/pB-DOX/ICG with different concentrations of ICG.


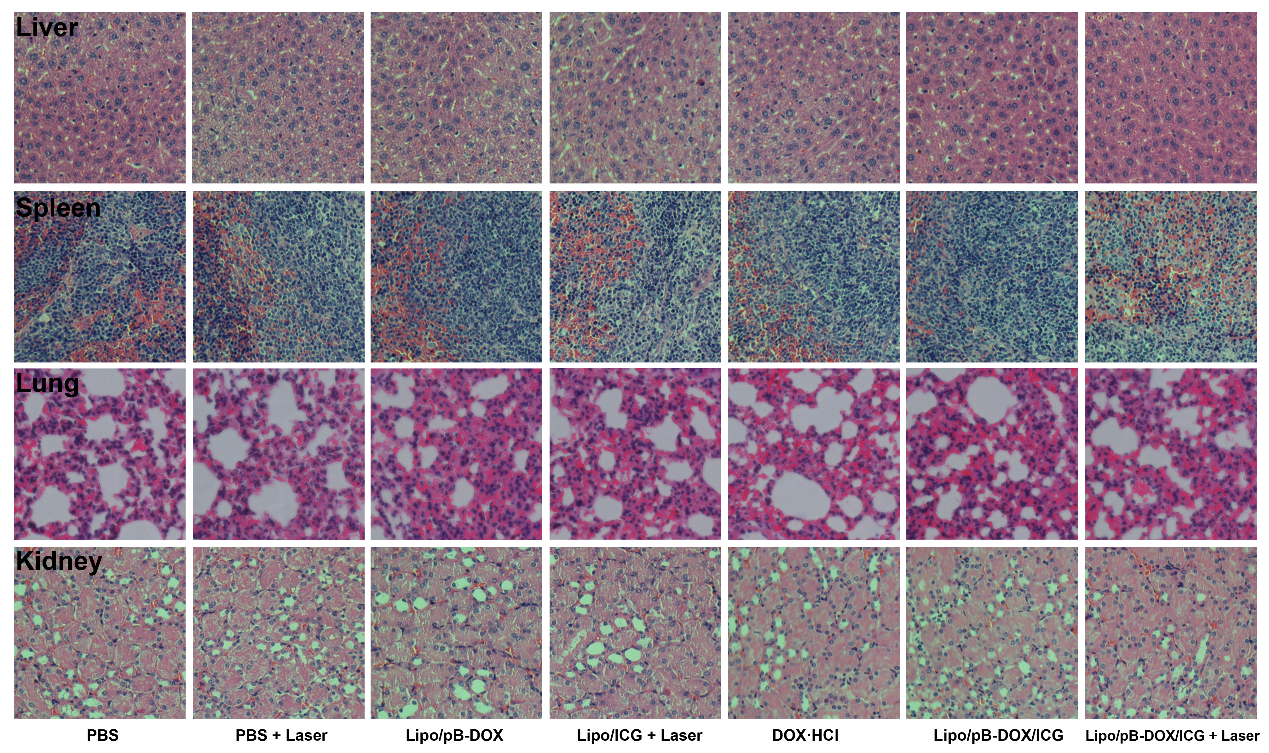


**Figure S8** Representative histological images of liver, spleen, lung and kidney samples from the treated mice. (n = 5 per group).
